# A network analysis of early arthropod evolution and the potential of the primitive

**DOI:** 10.1038/s41598-023-51019-x

**Published:** 2024-01-04

**Authors:** Agustín Ostachuk

**Affiliations:** 1 Museo de La Plata (MLP), Universidad Nacional de La Plata (UNLP), Buenos Aires, Argentina; 2 EVOLUTIO: A Research Center for Evolution and Development, Buenos Aires, Argentina

**Keywords:** Network topology, Evolutionary developmental biology

## Abstract

It is often thought that the primitive is simpler, and that the complex is generated from the simple by some process of self-assembly or self-organization, which ultimately consists of the spontaneous and fortuitous collision of elementary units. This idea is included in the Darwinian theory of evolution, to which is added the competitive mechanism of natural selection. To test this view, we studied the early evolution of arthropods. Twelve groups of arthropods belonging to the Burgess Shale, Orsten Lagerstätte, and extant primitive groups were selected, their external morphology abstracted and codified in the language of network theory. The analysis of these networks through different network measures (network parameters, topological descriptors, complexity measures) was used to carry out a Principal Component Analysis (PCA) and a Hierarchical Cluster Analysis (HCA), which allowed us to obtain an evolutionary tree with distinctive/novel features. The analysis of centrality measures revealed that these measures decreased throughout the evolutionary process, and led to the creation of the concept of *evolutionary developmental potential*. This potential, which measures the capacity of a morphological unit to generate changes in its surroundings, is concomitantly reduced throughout the evolutionary process, and demonstrates that the primitive is not simple but has a potential that unfolds during this process. This means for us the first empirical evolutionary evidence of our theory of evolution as a process of unfolding.

## Introduction

It is often thought that the primitive is simpler. This line of thought also maintains that the complex is generated from the simple by some process of self-assembly or self-organization, with diverse conceptual variations and tonalities. This concept was developed contemporaneously in the field of cybernetics. However, its origins can be traced back to the concept that a natural end must not only be organized, but must also be self-organized, set forth in Kant’s *Kritik der Urteilskraft* of 1790^1^. More rudimentarily, it can also be found in Greek materialism, developed first by Leucippus and Democritus, and then by Epicurus and Lucretius. They believed that everything is generated and can be generated by the spontaneous and fortuitous collision of elementary particles or atoms^2^.

The so-called principle of “self-organization” was proposed, as such, by William Ross Ashby in 1947^3^, and then underwent successive reformulations and further developments. Thus, for example, another cybernetician, Heinz von Foerster, formulated the principle of “order from noise” in 1960^4^. Then, the biophysicist Henri Atlan built on this concept to develop his principle of “complexity from noise” in 1972^5^. And, posteriorly, the chemist Ilya Prigogine (together with Isabelle Stengers) formulated a similar principle called “order out of chaos” in 1984^6^. The concept of self-organization assumes that order is produced spontaneously through a process of local interactions between parts from an initially disordered system. This process is facilitated by random disturbances (“noise”), which allow the system to explore different states until arriving and being attracted by a stable state called attractor.

Similarly, in the field of biology it is assumed that the evolution of organisms occurred through a random and aleatory process of organic modifications (in Darwin’s time morphological changes, currently genetic mutations), whose temporal accumulation enabled the generation of new species. What was added in this case, compared to the previous case, was a mechanism proposed by Darwin called “natural selection”^7^. According to this mechanism, only the species best adapted to their environments could survive (“the survival of the fittest”, according to Herbert Spencer), in a context where constant competition and “struggle for existence” reigned between species and individuals, with a clear influence and origin in liberal economy^8^.

This line of thought has notably influenced theories that attemp to explain the structure and form of the most primitive arthropods and crustaceans. There is a general historical consensus that the primitive arthropod or crustacean, *Urarthropod* or *Urcrustacean*, consisted of a simple organism. Thus, for example, Hessler and Newman^9^, considering Cephalocarida, Leptostraca (Malacostraca) and Notostraca (Branchiopoda) as the most primitive representatives of crustaceans, speculated that the most probable ancestor of crustaceans, based on morphological similarity, were the trilobites. Trilobites present a very simple general organization, characterized by serial homology and the presence of stenopodous limbs, that is, a serial, repetitive and linear morpho-topological structure. Something similar happened when the class Remipedia was discovered^10^. Their long bodies with many similar segments, without specialization, and their serially homonomous nonphyllopodous trunk limbs, were quickly interpreted as evidence of their primitiveness, and as the probable extant crustacean with the most primitive body plan^11^. Remipedia was considered the sister group of Cephalocarida, as we saw, another crustacean generally regarded as one of the most primitive^12^. Nowadays, the situation has changed and it is not considered a primitive crustacean, but rather a sister group of insects^13^. However, the general situation has not changed, and what is understood by primitive arthropod or crustacean, and the structural and morphological organization attributed to it, remains largely the same.

In this manner, both the principle of self-organization used by cybernetics, and the principle of natural selection used by biology, assume that the evident increase in the complexity of organisms throughout evolutionary history has occurred by a random and spontaneous process of interactions between simpler elements. In short, these theories assume and maintain that the complex can arise from the simpler. We have developed a whole theory of evolution based on the opposite principle: the complex cannot arise from the simpler^14^. We have already given the fundamental reason for founding this principle: the simpler does not have the necessary information to generate something more complex than itself. This theory maintains that the more complex must preexist the simpler, in potential or virtual form, to thus guarantee its formation. From a logical point of view, the proposition that the evolution from a unicellular organism to a primate, with its complex behaviors and mental activities, occurred by a mere process of random and spontaneous interactions between elementary parts, does not seem to have a solid argumentative basis. An organism is a teleological-purposeful formal agent, and all behavior presupposes and assumes the existence of an end. Consequently, teleology cannot be eliminated from nature or from the evolutionary process as a whole. On the contrary, teleology is the very driving force of this evolutionary process. This is another of the great pillars of our theory.

In a previous work, we had found evidence of our theory in the ontogenetic and metamorphic development of the crab^15^. In this work, we will present evidence for the theory in the early evolution of arthropods. To this end, a rigorous selection process was carried out to choose 12 representatives of the main groups that appeared in the early evolution of arthropods, i.e. fossils from the Burgess Shale, Orsten Lagerstätte, and extant crustaceans, considered by general consensus to be the most primitive of the phylum. From the detailed study of the external morphology of these organisms, network models were developed. Once these networks were obtained, several network measures (network parameters, topological descriptors and complexity measures) were evaluated, and a Principal Component Analysis (PCA) and Hierarchical Cluster Analysis (HCA) were carried out. This allowed the construction of a hypothetical evolutionary tree. This tree posed a scenario in which the evolution of arthropods is marked by an early bifurcation into two evolutionary branches: a left or large branch (networks greater than 400 nodes), and a right or small branch (networks less than 400 nodes). Predictably, *Yohoia* and *Canadaspis* were placed as primitive arthropods, as the originators of the left branch. Surprisingly, *Triops* (Branchiopoda: Notostraca) was placed as the successor of this branch, before other organisms usually considered more primitive, such as *Rehbachiella* and *Marrella*. On the other hand, *Branchinecta* (Branchiopoda: Anostraca) was placed as the originator of the right branch, before other organisms usually considered more primitive, such as *Waptia* and *Olenoides* (Trilobita). The evolutionary tree allowed, at the same time, to establish a rationale and to understand the behavior of the network measures. These measures had two basic patterns: ascending bifurcation and descending bifurcation. A large part of the network measures had an ascending bifurcation pattern, that is, they increased throughout the evolutionary process. These measures were characterized as extensive complexity measures in our previous work^15^. On the other hand, a group of network measures had a descending bifurcation pattern, decreasing their values throughout the evolutionary process. To investigate the meaning of these results in greater detail, the organisms were studied at the level of their morpho-topological structure by measuring different centrality measures. The results showed that most of these measures had a descending bifurcation pattern. Several of these centrality measures measure the degree of influence and power of an actor within the network. This allowed us to create the concept of *evolutionary developmental potential*, which measures the ability or capacity of a morphological structure to generate changes in its area of influence. Visualization of arthropod networks based on these measures showed that primitive organisms possessed a high concentration of evolutionary developmental potential throughout the body, while later organisms only had a high concentration in the head. Consequently, we come to the conclusion that the evolutionary developmental potential declines throughout the evolutionary process, determining a lower capacity to generate changes in later organisms. We interpret these empirical results as clear evidence for the theory of evolution as a process of unfolding that we have recently published^14^.

## Materials and methods

### Arthropod group selection criteria

The objective of the present work is to carry out a detailed and in-depth analysis of the early evolution of morphological-structural patterns of arthropods through the use of complex networks. This objective already imposes a limit on the possible number of networks included in the study, and the need to establish a selection criterion for the groups of arthropods to be included in the analysis. In this sense, we considered that the inclusion of 12 groups of arthropods was an adequate number to cover the wide spectrum and diversity of groups present in the phylum Arthropoda, the objective being precisely to carry out a network analysis of the evolution of structural patterns, and not carry out an extensive phylogenetic study. In the latter case, a larger sample is generally used, but perhaps does not have the possibility of tracking the deep structural changes that are occurring in the evolutionary process.

Another limit imposed on the work was the time scale. As the objective of the work was to study the early evolution of true arthropods, that is, arthropods with a fully arthrodized body organization, the appearance of the Upper stem-group Euarthropoda was established as the beginning of the time scale for our analysis^16,17^. In this manner, arthropods with a lobopodian/radiodontan-type body organization were left out of the analysis, that is, animals belonging to the groups Lobopodia and Radiodonta. The first important and most basal group of upper stem-group euarthropods, by general consensus, is formed by the so-called bivalved Cambrian arthropods^18,19^, which some authors include within the order Hymenocarina. Some of these authors, however, place this order later, practically as a preamble to the appearance of the clade Pancrustacea^20,21^. The second important basal group, which appears generally placed after the previous one, is the class Megacheira^18,19,21,22^. The next important group is represented by Artiopoda, which some authors consider as the stem-group Chelicerata, and whose best-known and famous Cambrian representative is the class Trilobita^18,19,21,22^. The next important group is the class Marrellomorpha^18,19,21^, which is generally considered a close relative of Artiopoda, and which some authors include together with it and Chelicerata in the clade Arachnomorpha^23^. However, other authors consider that these groups actually belong to the clade Mandibulata^24^. The last important group prior to the appearance of the clade Pancrustacea are the Cambrian Orsten arthropods or taxa^18,19^, so called because they were found in the Orsten Lagerstätte in Sweden. Some of the representatives of this group could even be found within the clade Pancrustacea^18,21^.

On the other hand, we also wanted to investigate the transition from extinct groups to extant groups, as has been done in several of the works cited above. In general terms, the instance of appearance of the first groups of extant arthropods is usually established with the appearance of the clade Pancrustacea^13,18,25,26^. Within this clade, there is a broad consensus to consider within the most basal groups the class Remipedia, the class Cephalocarida, the class Branchiopoda, in particular the order Anostraca and the order Notostraca, and the class Malacostraca, in particular the order Leptostraca^18,25,27^.

The choice of the particular representatives of each of the groups mentioned above was based on the fact of having sufficient information, in the form of monographs and extensive descriptions from primary sources, to make a robust and reliable reconstruction of their respective network models based on their morphological structure. This meant that in general the particular choices coincided, for example, with the most abundant genera existing in the Burgess Shale and Orsten Lagerstätte.

In this manner, the representatives of each of the selected groups were the following: *Canadaspis* (bivalved Cambrian arthropod), *Waptia* (bivalved Cambrian arthropod), *Yohoia* (Megacheira), *Olenoides* (Trilobita, Artiopoda), *Marrella* (Marrellomorpha), *Martinssonia* (Cambrian Orsten taxa), *Rehbachiella* (Cambrian Orsten taxa), *Branchinecta* (Branchiopoda: Anostraca), *Triops* (Branchiopoda: Notostraca), *Lightiella* (Cephalocarida), *Speleonectes* (Remipedia) and *Nebalia* (Malacostraca: Leptostraca). Table 1 details the list of arthropod groups included in the network analysis, and the primary bibliographic sources used for the detailed and thorough study of their morphological structures.

**Table 1:** Groups of extinct and extant arthropods selected for this work, and the corresponding references used for the study of their external morphology.

Group	References
*Canadaspis*	^99,100^
*Waptia*	^20,100,101^
*Yohoia*	^100–104^
*Olenoides*	^105,106^
*Marrella*	^100,101,107–110^
*Martinssonia*	^111^
*Rehbachiella*	^112^
*Branchinecta*	^113–116^
*Triops*	^117–122^
*Lightiella*	^123–125^
*Speleonectes*	^126–128^
*Nebalia*	^129–132^

### The arthropod network model

Arthropod networks were built according to the same principles as crustacean networks^15,28^, and can be considered an extension and broadening of these principles to the phylum Arthropoda. The distinctive characteristic of these groups is that they are formed by segments that are articulated in different ways with each other. This morphological pattern can be translated into network theory as nodes connected to each other by edges. In this manner, the different morphological units of arthropods, clearly identifiable, distinguishable and delimited, such as segments, articles, endites, exites, epipods and flagella, were considered nodes. The physical connections between these elements, articulated or non-articulated, were considered edges.

The detailed and systematic study of the morphology of arthropods, their different morphological units and connections between them, allows the creation of a blueprint or design plan of the network. This diagram is then translated into the computer language of network theory by preparing the adjacency matrix. This is a two-axis table composed of all the morphological units of the animal, and where the connection between two units is represented by the number 1 and the lack of connection by the number 0^29,30^. In this manner, the adjacency matrix has a dimension of $N \times N$, where *N* represents the total number of nodes in the network. This matrix, typically an object of *csv* file extension, is then used to build the actual network, typically an object of *graphml* file extension. The generation of the network from the adjacency matrix was carried out using the R programming language^31,32^, and the *igraph* network analysis package^33^. Networks were displayed and spatialized using the ForceAtlas 2 layout algorithm^34^, belonging to the network visualization software *Gephi*^35^. The adjacency matrices of the different groups of arthropods used in this work are provided as Supplementary Information.

### Network parameters

Once the networks were built, their basic characteristics were analyzed. For a first approximation to the characterization of their properties, the following network parameters were calculated: Node number, Edge number, Diameter, Radius, Average path length, Average degree, Average clustering coefficient, Density. Thus, for example, Average path length represents the arithmetic mean of all path lengths, which is the distance, the number of edges, that separates two nodes. Average degree is the arithmetic mean of all node degrees, which is the number of connections of a node. On the other hand, Density is the relationship between actual connections and all potential connections. All these parameters can be obtained with the *igraph* package.

### Topological descriptors

To carry out a deeper and more detailed analysis of arthropod networks, a battery of topological descriptors initially developed for the identification and discrimination of molecules and chemical structures was evaluated. Recently, these descriptors have been used for studies of morphological evolution, showing enormous analytical power to reveal topological properties of biological networks^15,28^.

These descriptors measure different topological properties of a network. Some descriptors consist of distance-based measures. Thus, for example, the Wiener index, the first topological descriptor developed, and perhaps the best known, consists of the sum of the distances between each pair of nodes in the network^36^. Another well-known distance-based descriptor is the Balaban J index. This index is obtained using the distance matrix, and calculating the distance sum for each node in the network, which is why they are also called distance degrees^37^. Other descriptors are entropy-based measures, such as the Bonchev index^38^ and the Bertz complexity index^39^. Another group of descriptors consists of eigenvalue-based measures. Within this group, there are the Estrada index, the Laplacian Estrada index, the Energy index and the Laplacian Energy index^40^. Finally, other descriptors are based on other graph-invariants, such as the Zagreb index^41^, the Randić connectivity index^42^, the Complexity index B and Normalized Edge complexity^43^. The latter is also called connectedness, and represents the ratio between the sum of all node degrees and the number of edges in the complete graph.

### Complexity measures

Some of the topological descriptors mentioned above can be considered complexity measures. However, many of them do not meet an important requirement for comparing complexity between different networks: they are not normalized complexity measures. Consequently, comparison between networks of different sizes is difficult. For this case, there is another package of complexity measures that are normalized. This group of measures assumes that the most complex networks have an intermediate number of edges, as this allows the existence of characteristic intricate internal structures, such as modular domains, hierarchical structures and specific functional regions^44^.

In this work, we used two of the three existing groups of complexity measures: Product measures and Entropy measures. Within the first group, there are Medium Articulation (*MAg*), Efficiency complexity (*Ce*) and Graph index complexity (*Cr*). *MAg* is basically the product between redundancy and mutual information, followed by normalization^44^. *Ce* measures how efficiently a network exchanges information using the inverse shortest path lengths^45^. *Cr* is based on the properties of the largest eigenvalue of the adjacency matrix, the index *r*^44^. All three measures were used in this work. On the other hand, within the second group, we evaluated the measure called Offdiagonal complexity (*OdC*). *OdC* measures diversity in the node-node link correlation matrix^46^.

### Principal component analysis (PCA)

Once all the networks parameters, topological descritptors and complexity measures were obtained, they were all used to carry out a Principal Component Analysis (PCA). The idea was to reveal hidden patterns derived from the structure and topology of the networks, and their relationship with the different topological and complexity measures. The PCA was carried out using the *prcomp* function of the R programming language. The visualization of the individuals and variables for the three main dimensions of the PCA was carried out using the *factoextra* package^47^.

### Hierarchical cluster analysis (HCA)

Hierarchical cluster analysis (HCA) is a clustering method for grouping objects based on their similarity. In agglomerative clustering, each observation is initially considered as a cluster (leaf), and then the most similar clusters are successively merged until a single cluster (root) is obtained. The result of a hierarchical clustering is a tree-based representation of the objects, i.e. a dendrogram. First, the data, containing the results obtained for the different topological and complexity measures, were scaled, using the *scale* function of the R programming language. Second, the dissimilarity matrix was calculated, to measure the degree of (dis)similarity between the networks. To do this, the *dist* function of the R programming language was used to compute the Euclidean distance between the networks. Finally, a linkage function was applied to group, from the distance information, pairs of objects into clusters based on their similarity. This is an iterative process that is repeated until all the objects are linked in a hierarchical tree. This was done with the *hclust* function of the R programming language, choosing the Ward method (*ward*.*D*2), which minimizes the total within-cluster variance. At each step, the clusters that are merged are those with the smallest between-cluster distance. The result of the HCA was visualized using the *factoextra* package, which allows visualizing the clustering with different graphical representations of the dendrogram (horizontal/vertical, circular, phylogenic). In order to compare results, another clustering method, Hierarchical K-Means Clustering, was tested. This combines the best of k-means clustering and hierarchical clustering. To do this, the *hkmeans* function from the *factoextra* package was used.

### Centrality measures

Centrality measures are measures that try to find the most important nodes in a network. This search does not have a single solution, because it will depend on what is understood and defined as important.

Two of the best-known centrality measures are Betweeness centrality and Closeness centrality. Betweeness centrality quantifies the number of times a node acts as a bridge in the shortest path between two other nodes^48^. Betweeness centrality then measures the potential of a node to control the flow of information in a network. On the other hand, the Closeness centrality of a node is the inverse of the sum of the shortest paths between that node and all the other nodes in the network^49^. In this manner, Closeness centrality measures the degree of closeness of a node to all the other nodes in the network.

Another very popular and well-known centrality measure is Eigenvector centrality^50^. Eigenvector centrality is a measure of the influence of a node within a network. This measure assigns relative scores to the nodes in the network following the idea that connections to high-scoring nodes are more important than connections to low-scoring nodes. A centrality measure related to Eigenvector centrality is Katz centrality^51^. Katz centrality measures the influence of a node taking into account all possible walks with all other nodes in the network. Connections with distant neighbors are penalized, however, with an attenuation factor. Another centrality measure related to Eigenvector centrality is PageRank centrality^52^. PageRank centrality also measures the importance of a node in a network taking into account the quantity and quality of its connections. The main difference is the existence of a scaling factor. Finally, another centrality measure related to Eigenvector centrality is Power centrality^53^. Power centrality can be considered as an extension of Eigenvector centrality, in which the addition of a parameter $\upbeta$ acts as an attenuation factor that allows greater flexibility of the measure. If Power centrality is interpreted following the idea that the power of a node depends on the power of the nodes to which it is connected, the magnitude of the attenuation factor regulates the extent of the recursive effect produced by the other nodes: a greater magnitude implies a slower decay.

Another interesting centrality measure is Decay centrality^54,55^. Decay centrality is a measure of the closeness of a node to all other nodes in the network. The difference with Closeness centrality is that the distance is penalized using a decay parameter $\updelta$. If the parameter value is low, nearby nodes have a higher weight than distant nodes. On the other hand, if the parameter value is high, there is no preference for nearby nodes and all distances are weighted equally. Another centrality measure used in this work, also related to Closeness centrality, is Information centrality^56^. Information centrality takes into account all possible paths between a pair of nodes and weights them relatively based on the information they contain. In this manner, the Information centrality of a node is an average of the information of all the paths that originate from that node.

One of the last centrality measures used in this work is Subgraph centrality^57^. Subgraph centrality measures a node’s participation in all subgraphs of a network, weighting them according to their size. Finally, a centrality measure related to Subgraph centrality is Communicability centrality^58^. Communicability centrality counts the number of walks using the matrix exponential, weighting walks according to their length by a penalization factor. The total communicability of a node measures how well that node communicates with the other nodes in the network. Communicability centrality is a measure of the ease of sending information over the network.

Centrality measures were calculated using the *igraph*^33^ and *netrankr*^59^ packages. On the other hand, the visualization of the networks with the different centrality measures was carried out using the *tidygraph*^60^ and *ggraph*^61^ packages.

## Results

### Arthropod networks

**Figure 1:**
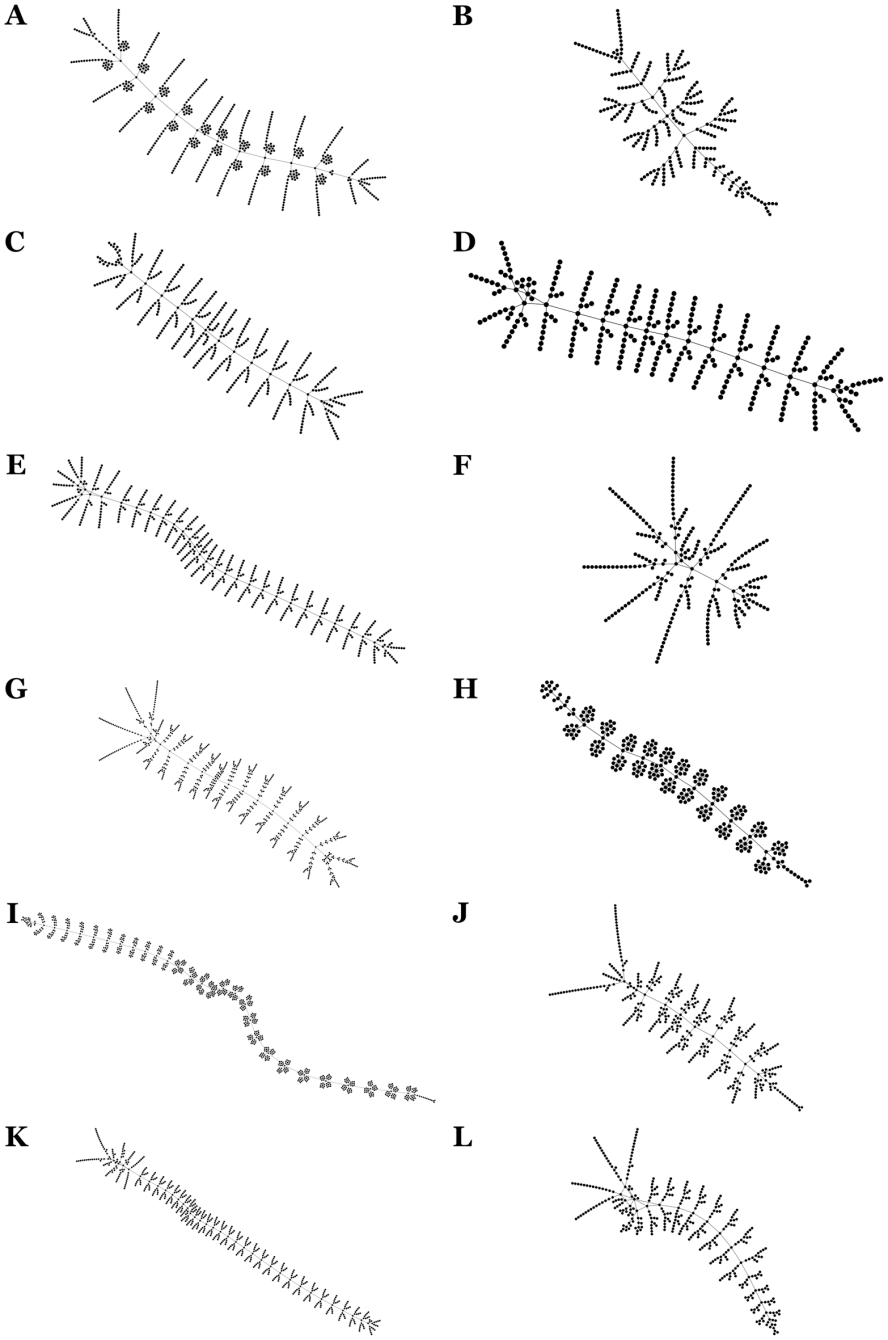
Arthropod networks. The external morphology of arthropods was abstracted and codified into the language of network theory. The resulting networks were displayed and spatialized using the ForceAtlas 2 layout algorithm of the network visualization software *Gephi*. Node size is the same in all cases. The difference in the size of nodes in the figure is due to a difference in scale. These are the networks with their respective numbers of nodes in parentheses: (**A**) *Canadaspis* (601), (**B**) *Waptia* (276), (**C**) *Yohoia* (457), (**D**) *Olenoides* (297), (**E**) *Marrella* (569), (**F**) *Martinssonia* (250), (**G**) *Rehbachiella* (747), (**H**) *Branchinecta* (288), (**I**) *Triops* (929), (**J**) *Lightiella* (383), (**K**) *Speleonectes* (624), (**L**) *Nebalia* (376).

The study of the external morphology of the 12 groups of arthropods selected for analysis allowed their abstraction in the language of network theory, that is, in the form of nodes connected by edges. The variation in the size of the networks was very wide, ranging between 250 and 929 nodes. Figure 1 shows the topological structure of the networks and indicates the size of each of them.

### Principal component analysis (PCA)

With the idea of revealing hidden patterns derived from the structure and topology of the networks, and their relationship with the different topological and complexity measures, we carried out a PCA using the three groups of network measures: network parameters, topological descriptors and complexity measures. This analysis revealed surprising and interesting behaviors and evolutionary processes, which will become clearer with the deeper analyzes carried out later (Hierarchical Clustering and Centrality measures).

It is worth mentioning that the result of the PCA seems to be very robust, since it was also carried out only with the topological descriptors, and the results obtained were very similar, almost identical, to those obtained in the PCA analyzed below.

#### Networks

**Figure 2:**
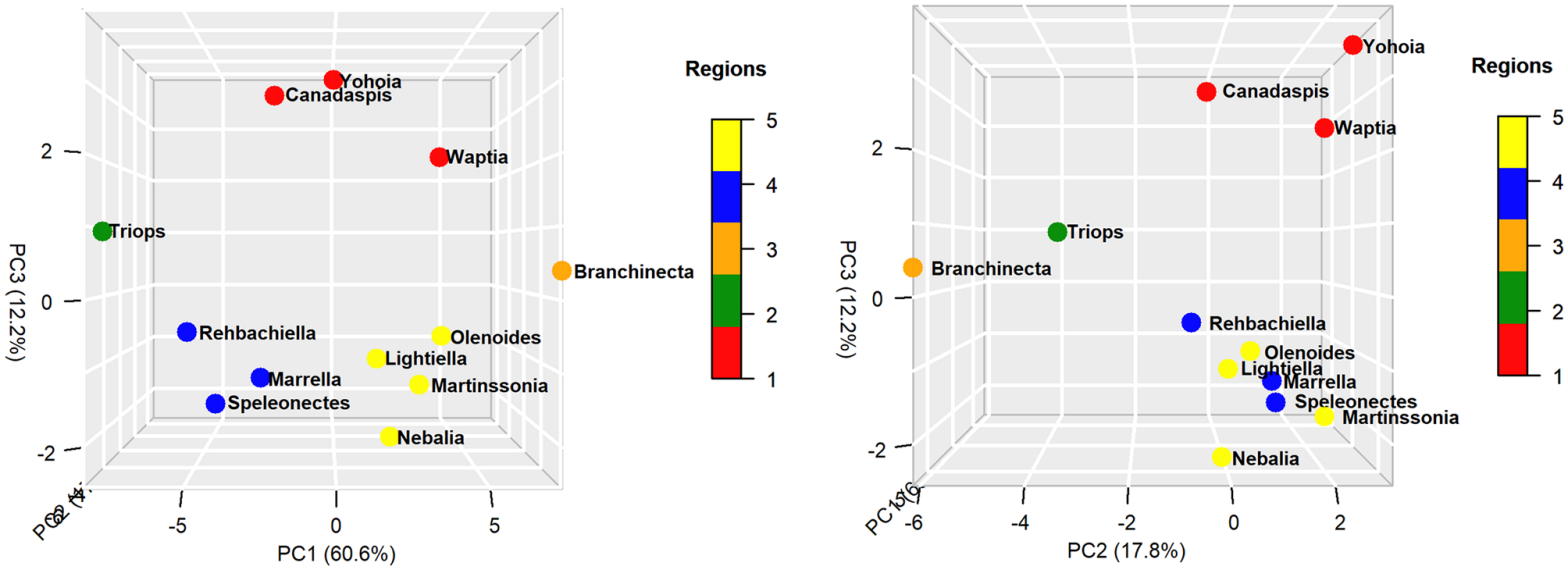
Principal component analysis (PCA). Distribution of arthropod networks in the three-dimensional space formed by the first three principal components, seen in two different orientations. Dot color varies according to the region to which each group belongs (see sidebar).

From a topological point of view, the PCA appeared to distribute the networks into 5 distinct topological regions (Fig. 2). A first region (Region 1) was characterized by being located in the positive zone of dimension 2 and 3, occupying approximately the central position with respect to dimension 1. The networks corresponding to *Canadaspis*, *Yohoia* and *Waptia* were located in this region, the latter being located in the positive zone of dimension 1. A second region (Region 2) was characterized by being located in the negative zone of dimension 1 and 2, occupying approximately the central region with respect to dimension 3. The network corresponding to *Triops* was found in this region. A third region (Region 3) was characterized by being located in the positive zone of dimension 1 and the negative zone of dimension 2, also occupying approximately the central region with respect to dimension 3. The network corresponding to *Branchinecta* was found in this region. Region 3 was then located in the opposite zone with respect to dimension 1 than Region 2. In turn, Region 2 and 3 were located in the opposite zone with respect to dimension 2 than Region 1. For its part, a fourth region (Region 4) was characterized by being located in the negative zone of dimension 1 and 3, and the positive zone of dimension 2. The networks corresponding to *Rehbachiella*, *Marrella* and *Speleonectes* were found in this region. Finally, a fifth region (Region 5) was characterized by being located in the positive zone of dimension 1 and 2, and the negative zone of dimension 3. The networks corresponding to *Olenoides*, *Martinssonia*, *Nebalia* and *Lightiella* were found in this region. Region 5 was then located on the opposite zone with respect to dimension 1 than Region 4, so that Region 5 shared the positive zone of dimension 1 with Region 3, and Region 4 shared the negative zone of dimension 1 with Region 2. For their part, Region 4 and 5 occupied the opposite region with respect to dimension 3 than Region 1, while they coincided with respect to dimension 2.

The richest information seemed to be found in dimensions 1 and 3 of the PCA (Fig. 2, left). Dimension 1 explained 60.6% of the variation, while dimension 3 explained 12.2% of the variation. On the other hand, the first three dimensions together explained 90.6% of the variation. Figure 2 (left) seems to show a scenario where a group of originary arthropods, formed by *Canadaspis*, *Yohoia*, and perhaps also *Waptia*, gave rise to two large evolutionary branches: (1) left or large branch (networks larger than 400 nodes), led by *Triops*, and formed by the largest arthropods (*Rehbachiella*, *Marrella*, *Speleonectes*); and (2) right or small branch (networks smaller than 400 nodes), led by *Branchinecta*, and formed by the smallest arthropods (*Olenoides*, *Martinssonia*, *Nebalia*, *Lightiella*). According to the results of this analysis, dimension 3 would be determining the temporality of the evolutionary process, while dimension 1 would be determining the spatiality/diversification of this process.

If this evolutionary scenario is correct, two quite surprising events stand out and are revealed as novel with respect to the currently existing information: (1) the existence of a very early bifurcation of arthropods, and (2) a prominent and decisive role of *Triops* and *Branchinecta* in this early process of bifurcation. These results and evidence, if verified, would provide a novel and hitherto unexplored view of early arthropod evolution. The orders Anostraca and Notostraca, belonging to class Branchiopoda, are generally considered primitive crustaceans, but clearly not at the level of basal arthropods. This privileged position of *Triops*, as a representative of Notostraca, and *Branchinecta*, as a representative of Anostraca, in the early evolution of arthropods is reaffirmed and highlighted by dimension 2 of the PCA (Fig. 2, right). Basically, this dimension separates these two groups from the rest of the other arthropod groups. Later, we will try to explain the structural cause of this difference, as well as the structural cause that characterizes the most primitive arthropods according to the present analysis: *Canadaspis*, *Yohoia* and *Waptia*. In other words, the structural changes that would be taking place along dimensions 2 and 3 of this analysis will be investigated. In the following section, we will analyze the contribution of the different network measures to the first three dimensions of the PCA.

#### Network measures

**Figure 3:**
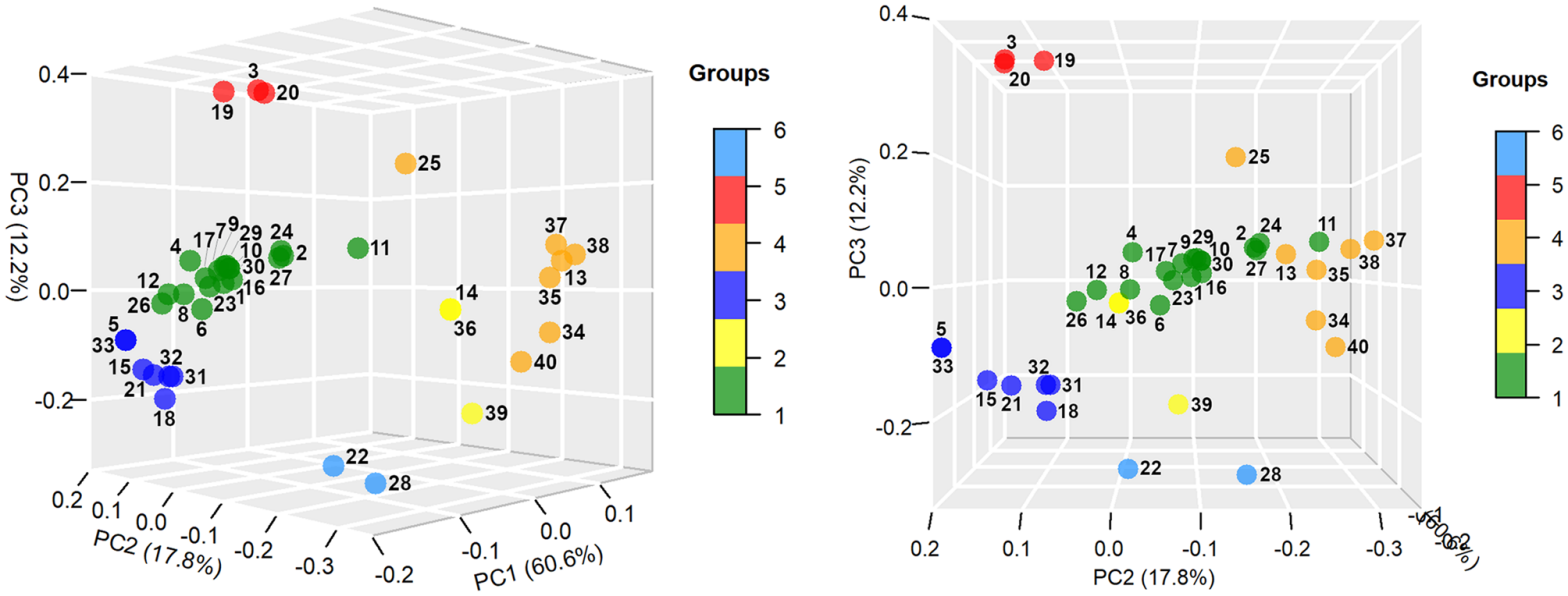
Principal component analysis (PCA). Distribution of network measures in the three-dimensional space formed by the first three principal components, seen in two different orientations. Dot color varies according to the group to which each measure belongs (see sidebar). Network measures: (1) Wiener index, (2) Harary index, (3) Balaban J index, (4) Mean distance deviation, (5) Compactness, (6) Eccentricity, (7) Centralization, (8) Average distance, (9) Konstantinova index, (10) Zagreb index 1, (11) Zagreb index 2, (12) Randić connectivity index, (13) Complexity index B, (14) Normalized edge complexity, (15) Bonchev index 1, (16) Bonchev index 2, (17) Bertz complexity index, (18) Radial centric information index, (19) Balaban-like information index 1, (20) Balaban-like information index 2, (21) Graph vertex complexity index, (22) Edge equality, (23) Information layer index, (24) Estrada index, (25) Laplacian Estrada index, (26) Energy, (27) Laplacian energy, (28) Spectral radius, (29) Nodes, (30) Edges, (31) Diameter, (32) Radius, (33) Average path length, (34) Average degree, (35) Average clustering coefficient, (36) Density, (37) *MAg*, (38) *Ce*, (39) *Cr*, (40) *OdC*.

An important number of network measures (Group 1) contributed mainly to the negative axis of dimension 1, with different degrees of contribution to the negative axis of dimension 2, while they contributed very little to dimension 3 (region: PC1 negative, PC2 center to negative, PC3 center) (Fig. 3). A subgroup of network measures were almost aligned with the negative axis of dimension 1 (Randić connectivity index, Average distance, Mean distance deviation, Energy), while another subgroup contributed moderately to the negative axis of dimension 2 (Nodes, Edges, Wiener index, Centralization, Eccentricity, Zagreb index 1, Bertz complexity index, Bonchev index 2, Konstantinova index, Information layer index), and a third subgroup contributed highly to the negative axis of dimension 2 (Estrada index, Harary index, Laplacian energy, Zagreb index 2).

The group located in the opposite region to the previous group (Group 2) was characterized by contributing to the positive axis of dimension 1, and contributing very little to the other two dimensions (region: PC1 positive, PC2 center, PC3 center). The network measures Density, Normalized edge complexity and the complexity measure *Cr* were found in this group. The latter, unlike the other two, not only contributed to the positive axis of dimension 1, but also to the negative axis of dimension 3. Groups 1 and 2 were the groups that were projected and essentially determined dimension 1 of the PCA, in its negative and positive direction, respectively.

Another group of network measures (Group 3) not only contributed to the negative axis of dimension 1, but also to the positive axis of dimension 2 and the negative axis of dimension 3 (region: PC1 negative, PC2 positive, PC3 negative). These are the network measures Diameter, Radius, Average path length, Graph vertex complexity, Compactness, Bonchev index 1 and Radial centric information index.

The group that was somehow located in the opposite region to the previous group (Group 4) was characterized by contributing largely to the negative axis of dimension 2, with a variable contribution to the positive axis of dimension 1, and little contribution to dimension 3 (region: PC1 center to positive, PC2 negative, PC3 center). The following network measures could be found in this group: Average degree, Average clustering coefficient, Complexity index B, *MAg*, *Ce*, *OdC*. Most of the complexity measures used were found in this group. Groups 3 and 4 were the groups that were projected and essentially determined dimension 2 of the PCA, in its positive and negative direction, respectively.

A very important group (Group 5), for reasons that we will see and analyze later, were the network measures characterized by contributing to the positive axis of dimension 2 and 3 (region: PC1 center, PC2 positive, PC3 positive). The network measures Balaban J index, Balaban-line information index 1 and Balaban-like information index 2 were found in this group. The Balaban-like information index 1, unlike the other two, had a moderate contribution to the negative axis of the dimension 1.

The group that was somehow located in the opposite region to the previous group (Group 6) was characterized by contributing essentially to the negative axis of dimension 3, with a very low contribution to dimension 1 and 2 (region: PC1 center, PC2 center, PC3 negative). The network measures Edge equality and Spectral radius were found in this group. The second had a moderate contribution to the negative axis of dimension 2. Groups 5 and 6 were the groups that were projected and essentially determined dimension 3 of the PCA, in its positive and negative direction, respectively.

### Hierarchical cluster analysis (HCA)

The PCA provided very valuable information and seemed to reveal an evolutionary pattern determined by dimension 3 as temporality and dimension 1 as spatiality/diversification. In this evolutionary pattern, *Canadaspis*, *Yohoia* and *Waptia* appeared as the most primitive arthropods, followed surprisingly by *Triops* and *Branchinecta*, inaugurating two well-differentiated evolutionary lineages. To confirm or refute this interpretation of the PCA, a Hierarchical Cluster Analysis (HCA) was carried out based on the results of the network measures. This involved first scaling these results, then calculating the degree of (dis)similarity between the networks, and finally grouping the networks into clusters according to their degree of similarity, following an agglomeration method. The hierarchical clustering used in this work is equivalent to the one known as Unweighted Pair-Group Method using Arithmetic mean (UPGMA), although using *ward*.*D*2 as agglomeration method. The difference between the two is that the branch lengths change.

**Figure 4:**
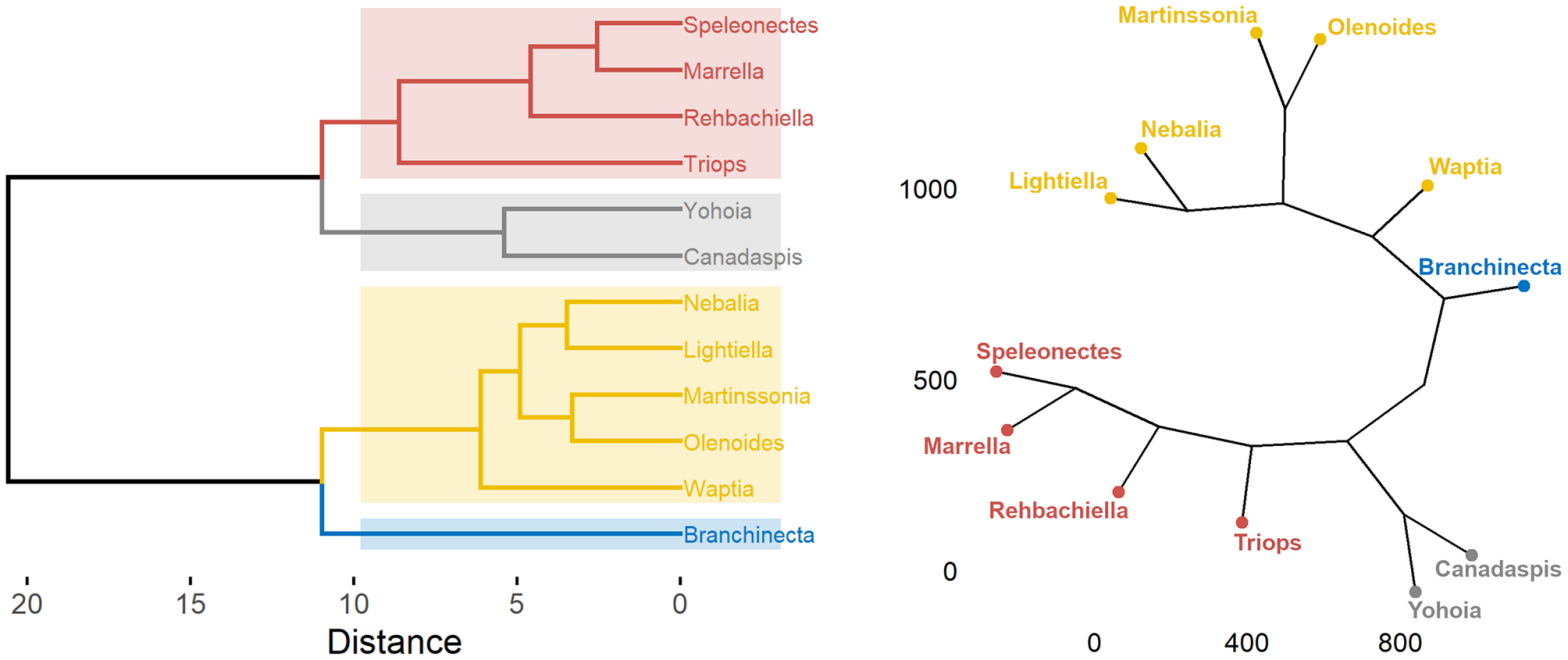
Hierarchical Clustering Analysis (HCA). Horizontal (left) and phylogenic (right) dendrograms of arthropod networks, carried out by agglomerative hierarchical clustering and using Ward’s method (*ward.D2*). Dendrograms were divided into 4 clusters, shown in different colors, based on the degree of similarity.

The result of the Hierarchical Cluster Analysis (HCA) is shown in the dendrograms in Fig. 4, using two different types of dendrograms, *rectangle* (left) and *phylogenic* (right). The correlation between the cophenetic distances and the original distance data (cophenetic correlation coefficient) was 0.685. The use of another clustering method, Hierarchical K-Means Clustering, which combines the best of k-means clustering and hierarchical clustering, gave exactly the same results. All this is an indication that the results are solid and robust.

The result of the HCA largely confirms the qualitative analysis performed on the PCA, with some interesting differences. First, it places the origin of arthropods at an intermediate point between the pair *Canadaspis*-*Yohoia* and *Branchinecta*. Second, *Branchinecta* is placed as the originator of what we have called the right or small branch, while *Canadaspis* and *Yohoia*, their common ancestor to be more precise, are placed as originators of the left or large branch. Third, *Waptia* then appears as the first exemplar of the branch originated by *Branchinecta*, while *Triops* appears as the first exemplar of the branch originated by *Canadaspis*-*Yohoia*. In this manner, the statistical analysis carried out by means of hierarchical clustering locates the origin of arthropods not so much in *Canadaspis* and *Yohoia*, as the PCA result suggested, but in an ancestor of these two and of *Branchinecta*, probably more similar to the latter than the first two. As a consequence of this, *Waptia* is still considered a primitive group, but posterior and derived from *Branchinecta*. The placement of *Canadaspis* and *Yohoia* as two of the most primitive arthropods is not surprising, and it is what is usually believed of these Burgess Shale specimens. However, the positions of *Branchinecta* and *Triops* close to the point of origin, and giving rise to two early evolutionary branches of arthropods (in the case of *Triops* following *Canadaspis* and *Yohoia*), is an intriguing and novel result. In subsequent sections, we will see what morphological and topological characteristics would explain the primitiveness of these organisms, and what modifications of these characteristics would occur throughout the evolutionary process.

**Figure 5:**
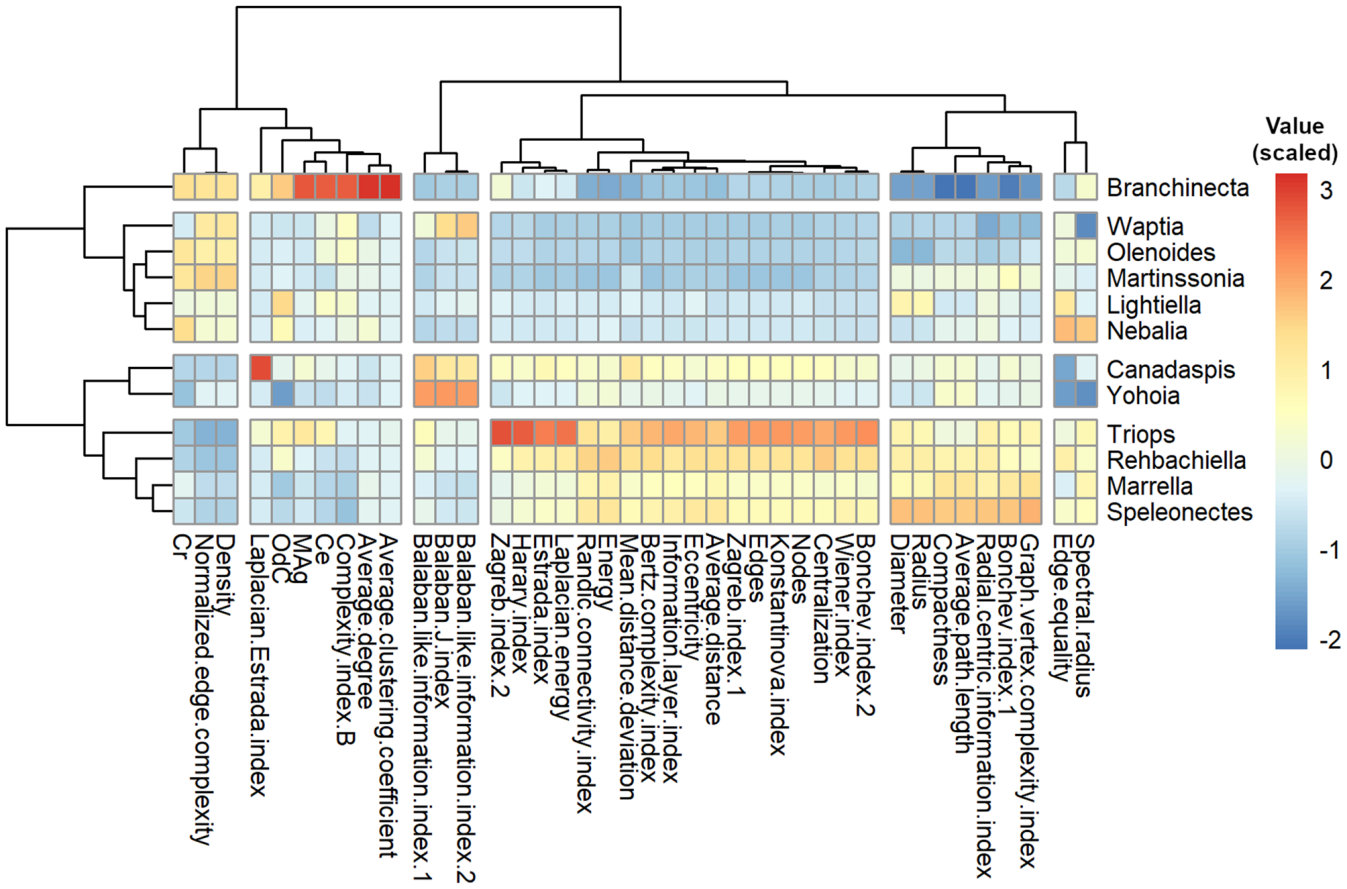
Hierarchical Clustering Analysis (HCA). Heatmap of arthropod networks and network measures, carried out by agglomerative hierarchical clustering and using Ward’s method (*ward.D2*). The dendrogram of arthropod networks was divided into 4 clusters, while the dendrogram of network measures was divided into 6 clusters, based on the degree of similarity. Color represents the value of the scaled measures (see side scale).

The representation of the HCA in the form of a heatmap (Fig. 5) provides a more visual analysis of the results analyzed above, while also providing a hierarchical clustering of the network measures. We can see in this format that there is a clear topological difference between the networks of organisms belonging to the right or small branch, and those belonging to the left or large branch. For example, while the networks of the right branch present negative values for the network measures that we had grouped in Group 1 in the PCA, the networks of the left branch present positive values for these measures. Practically the same happens with the network measures that we had grouped into Group 3 in the PCA. On the other hand, the opposite occurs with the measures grouped in Group 2: while the networks of the right branch present positive values for these network measures, the networks of the left branch present negative values.

The HCA carried out on the network measures coincides exactly with the division of groups that we had done qualitatively in the PCA. The same 6 groups defined in the PCA are the same 6 clusters into which the hierarchical clustering can be divided, each of them being formed by the same network measures as those previously defined (with the exception of Laplacian Estrada index which we had not included in our so-called Group 4). The two large clusters into which this hierarchical clustering can be divided basically divides the complexity measures from the topological descriptors, with the network parameters distributed between both clusters. On the other hand, some topological descriptors were located in the cluster where the complexity measures were found. In this manner, the network parameters Density, Average degree and Average clustering coefficient, and the topological descriptors Normalized edge complexity, Complexity index B and Laplacian Estrada index, were grouped in this cluster. The congruence between the HCA results and the results of the PCA is an indication that the results are solid, reliable and robust.

### Network measures

Once the hypothetical tree corresponding to the early evolution of arthropods has been obtained, we will now analyze in detail the results obtained for the different network measures used in this work: network parameters, topological descriptors and complexity measures. To do this, we decided to order the groups of arthropods according to the result of the hierarchical clustering, for which the most primitive organisms were placed in the center of the figures, on the right the organisms belonging to the right or small evolutionary branch, and on the left the organisms belonging to the left or large evolutionary branch. Thus, the arthropod groups were ordered as follows (from left to right): *Speleonectes*, *Marrella*, *Rehbachiella*, *Triops*, *Canadaspis*, *Yohoia*, *Branchinecta*, *Waptia*, *Olenoides*, *Martinssonia*, *Nebalia* and *Lightiella*.

#### Network parameters

Ordered according to the early evolutionary bifurcation proposed by the PCA and the HCA, we detected two basic patterns regarding the behavior of the network parameters, which would later be repeated in the other network measures. The first pattern, which we could call *ascending bifurcation*, is characterized by having the minimum value at the hypothetical evolutionary origin and by increasing on both sides of the origin along the right and left evolutionary branches. The second pattern, which we could call *descending bifurcation*, presents the maximum value at the hypothetical evolutionary origin and decreases on both sides of the origin along the right and left evolutionary branches.

**Figure 6:**
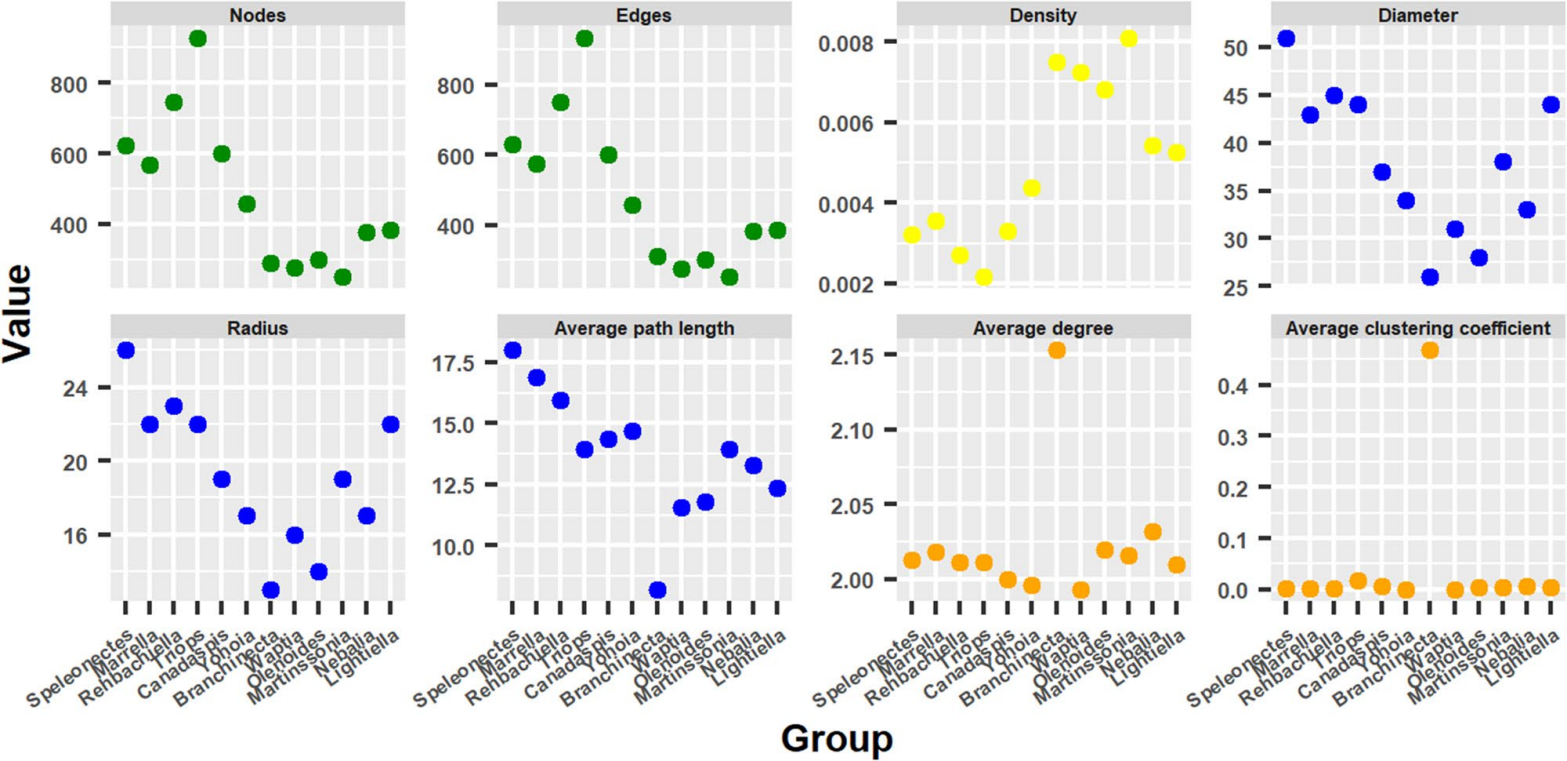
Network parameters. Behavior of network parameters ordering the arthropod networks according to the result of the hierarchical clustering: right branch from the center to the right (from *Branchinecta* to *Lightiella*), and left branch from the center to the left (from *Yohoia* to *Speleonectes*). Color represents the group to which each network parameter belongs.

The network parameters that behaved according to the first pattern were clearly Diameter, Radius and Average path length, all three members of Group 3 (blue) (Fig. 6). We could also include Nodes and Edges (Group 1, green) within this group, with the difference that in these network parameters the left branch ascends to *Triops* and then descends. On the other hand, the network parameters that behaved according to the second pattern were Density (Group 2, yellow), which had a slight rise in the left branch from *Triops* onwards, and Average degree and Average clustering coefficient (Group 4, orange), despite the fact that these parameters were only higher in *Branchinecta*. As can be seen in the figure, the size of the arthropod networks ranged from about 300 nodes (for example, in *Branchinecta*) to about 900 nodes (in *Triops*).

#### Topological descriptors

**Figure 7:**
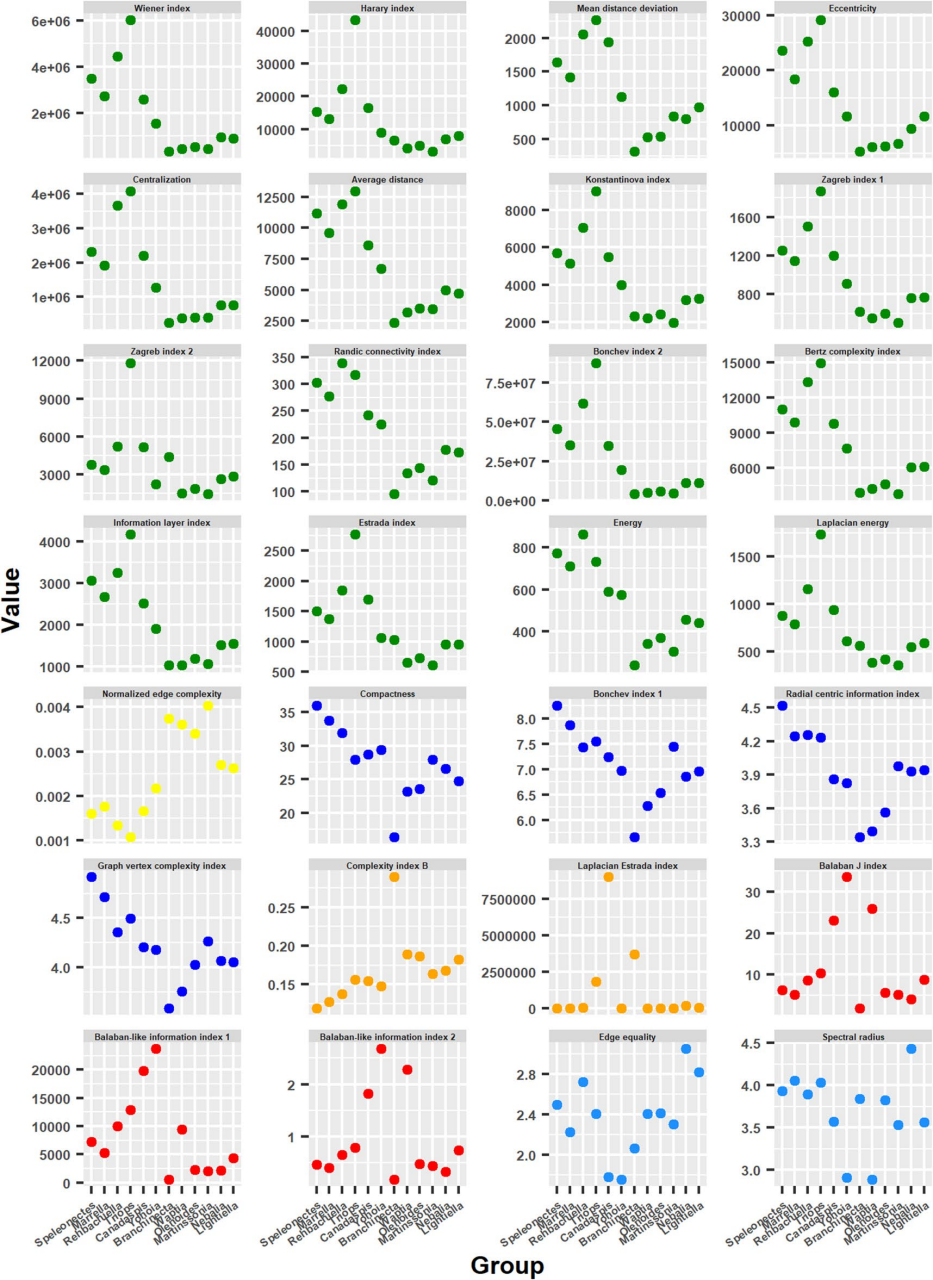
Topological descriptors. Behavior of topological descriptors ordering the arthropod networks according to the result of the hierarchical clustering: right branch from the center to the right (from *Branchinecta* to *Lightiella*), and left branch from the center to the left (from *Yohoia* to *Speleonectes*). Color represents the group to which each topological descriptor belongs.

As occurred with the network parameters, the topological descriptors showed in their evolution the two basic patterns mentioned above: *ascending bifurcation* and *descending bifurcation* (Fig. 7).

The topological descriptors belonging to Group 1 (green) largely showed the ascending bifurcation pattern: having their minimum values in the central zone, typically in *Branchinecta*, their values ascended in both evolutionary branches. The rise was more pronounced in the left branch, while in general it began to decline from *Triops* onwards, with some exceptions such as Randić connectivity index and Energy. The rise in the right branch was more moderate and variable, with relatively high rises such as in Mean distance deviation, Eccentricity, Randić connectivity index and Energy; and relatively low rises as in Wiener index, Harary index, Centralization and Bonchev index 2. The topological descriptors of Group 3 (blue) behaved similarly to those of Group 1. The most important difference is that in this case there was no decrease in the left branch, and the rise in the right branch was more pronounced. We could also include those of Group 6 (light blue) within the topological descriptors that evolved according to the ascending bifurcation pattern.

On the other hand, the topological descriptors of Groups 2 (yellow), 4 (orange) and 5 (red) evolved following the descending bifurcation pattern. This pattern was especially notable in the topological descriptors of Group 5 (Balaban J index, Balaban-like index 1 and Balaban-like index 2), a group that showed the peculiarity and rarity that one of the groups in which high values were expected of these measures (*Branchinecta*) obtained very low values. Something similar happened with Laplacian Estrada index (Group 4), in which *Yohoia* obtained much lower values than expected. For its part, Normalized edge complexity (Group 2) had an almost identical behavior to Density, another member of this group. The maximum value varied in the different groups. The maximum value in Group 5 was obtained by *Yohoia*. The maximum value in Group 2 was obtained by *Branchinecta*. Meanwhile, the maximum value in Group 4 was obtained by *Branchinecta* (Complexity index B) and *Canadaspis* (Laplacian Estrada index).

#### Complexity measures

**Figure 8:**
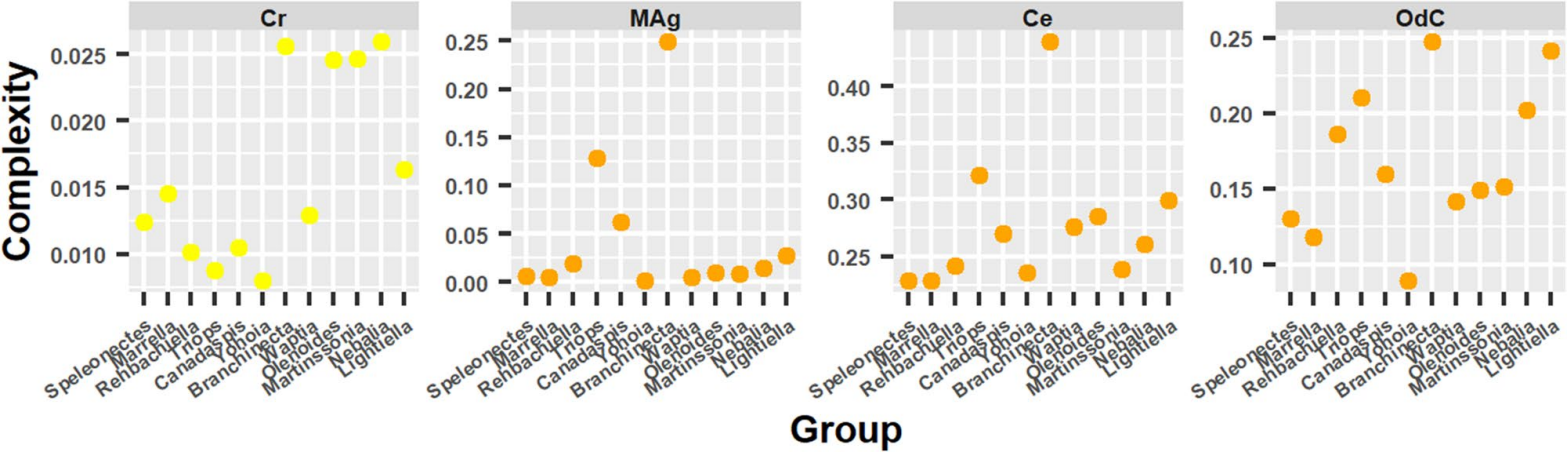
Complexity measures. Behavior of complexity measures ordering the arthropod networks according to the result of the hierarchical clustering: right branch from the center to the right (from *Branchinecta* to *Lightiella*), and left branch from the center to the left (from *Yohoia* to *Speleonectes*). Color represents the group to which each complexity measure belongs.

The complexity measures did not have as clear a behavior as in the previous cases, but they could still be included in one of the two characteristic evolutionary patterns (Fig. 8).

Perhaps the measure that best adapted to one of these two patterns was *MAg* (Group 4), which gave high values for *Canadaspis*, *Branchinecta* and *Triops*, but low for *Yohoia*, a pattern similar to that shown by the Laplacian Estrada index. The same occurred with the complexity measure *Ce* (Group 4), which gave high values only for *Branchinecta*, and intermediate values for *Triops*, a pattern similar to that shown by Complexity index B. In this manner, we could affirm that these two measures had a descending bifurcation pattern, which then seems to be the characteristic evolutionary pattern of the measures of Group 4, as also seems to be the case for the measures of Group 5. The complexity measure *OdC*, also of Group 4, does not seem to share this characteristic with its group, as it essentially showed an ascending bifurcation pattern, with one exception: *Branchinecta* gave a high value instead of a low one. At the same time, the left branch descended again from *Triops* onwards as occurred with various topological descriptors that showed this pattern, especially those of Group 1.

On the other hand, the complexity measure *Cr*, belonging to Group 2, had a behavior that was difficult to classify. We could say that it essentially showed an ascending bifurcation evolutionary pattern, with the exception that the measure gave high values for almost all members of the right branch. The value obtained by *Branchinecta* was very high than expected in the case of an ascending bifurcation pattern. The other two members of this group, Density and Normalized edge complexity, were more easily categorized in the descending bifurcation pattern. The characteristic that *Cr* shared with the other members of Group 2 was that it obtained much higher values for the right branch than for the left branch.

### Centrality measures

We have already analyzed the behavior of network measures and the possible early evolutionary process of arthropods, characterized by the presence of a primitive group of arthropods, from which two evolutionary branches arise, right and left. Now we will begin to try to unravel what this evolutionary process consists of: what structural changes occur in arthropod networks that explain progress along these evolutionary lines. We will do this by investigating the behavior of centrality measures.

**Figure 9:**
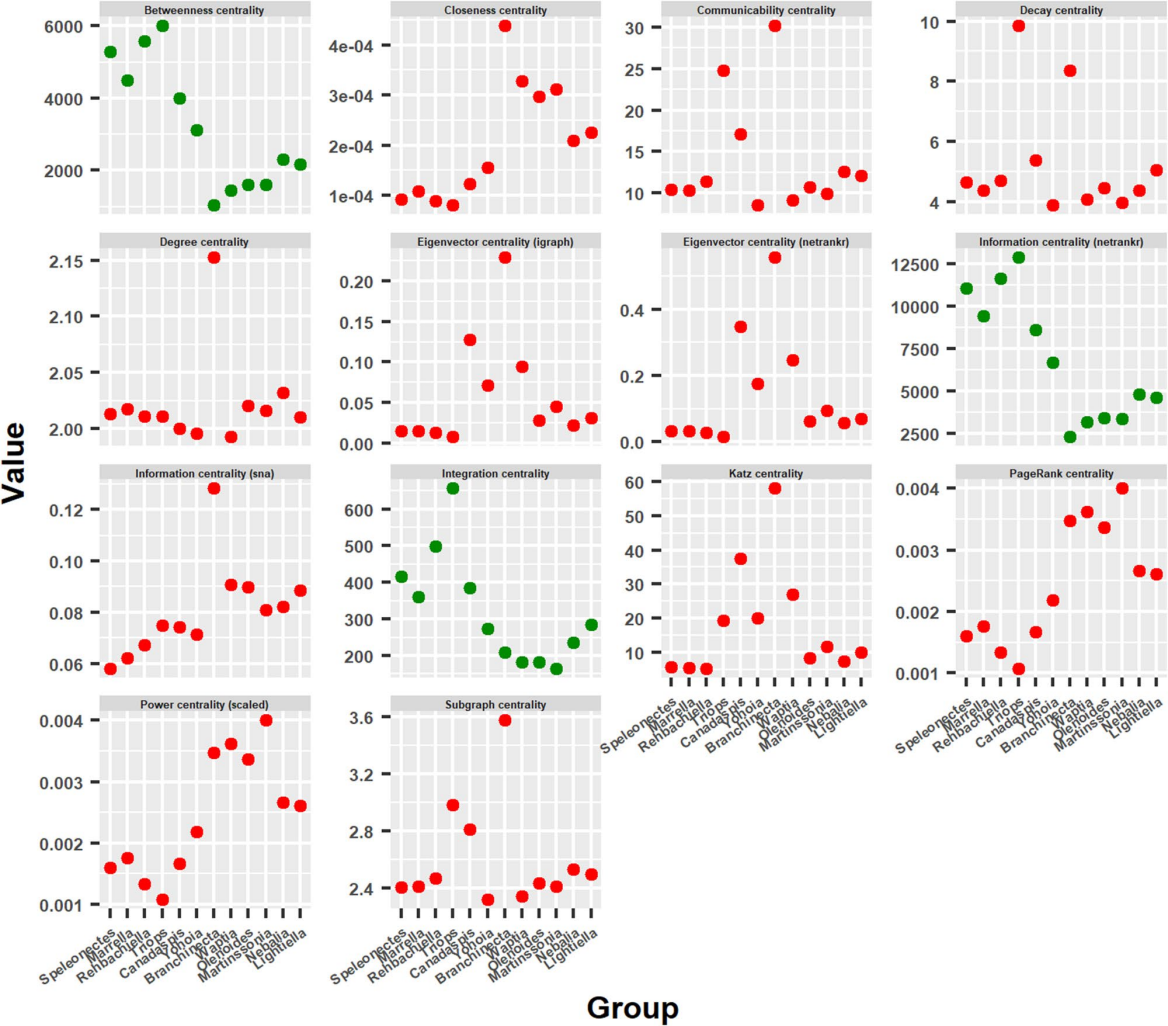
Centrality measures. Behavior of centrality measures’ mean values ordering the arthropod networks according to the result of the hierarchical clustering: right branch from the center to the right (from *Branchinecta* to *Lightiella*), and left branch from the center to the left (from *Yohoia* to *Speleonectes*). Most centrality measures had a descending bifurcation pattern (red), while a few exceptions had an ascending bifurcation pattern (green).

Interestingly, in contrast to what happened with the topological descriptors, most of the centrality measures had a descending bifurcation pattern (red) (Fig. 9). The few exceptions were Betweenness centrality, Information centrality (*netrankr*) and Integration centrality, which had an ascending bifurcation pattern (green) very similar to that obtained by Group 1 of network measures. Within the centrality measures with a descending bifurcation pattern, there were different specific variants. Thus, for example, PageRank centrality and Power centrality (scaled) had a very similar pattern to that obtained by Density and Normalized edge complexity. On the other hand, Information centrality (*sna*) had a very similar pattern to Complexity index B.

The results obtained with the centrality measures are very interesting, since they are indicating the presence of a property or characteristic in the most primitive arthropods that is lost throughout the evolutionary process. Among these centrality measures, Eigenvector centrality, Katz centrality, Power centrality and PageRank centrality stand out, since they all derive from the same general basic principle. A general basic principle that will be important for the results and conclusions of our work. We will now study in detail what happens with the centrality measures throughout the evolutionary process.

**Figure 10:**
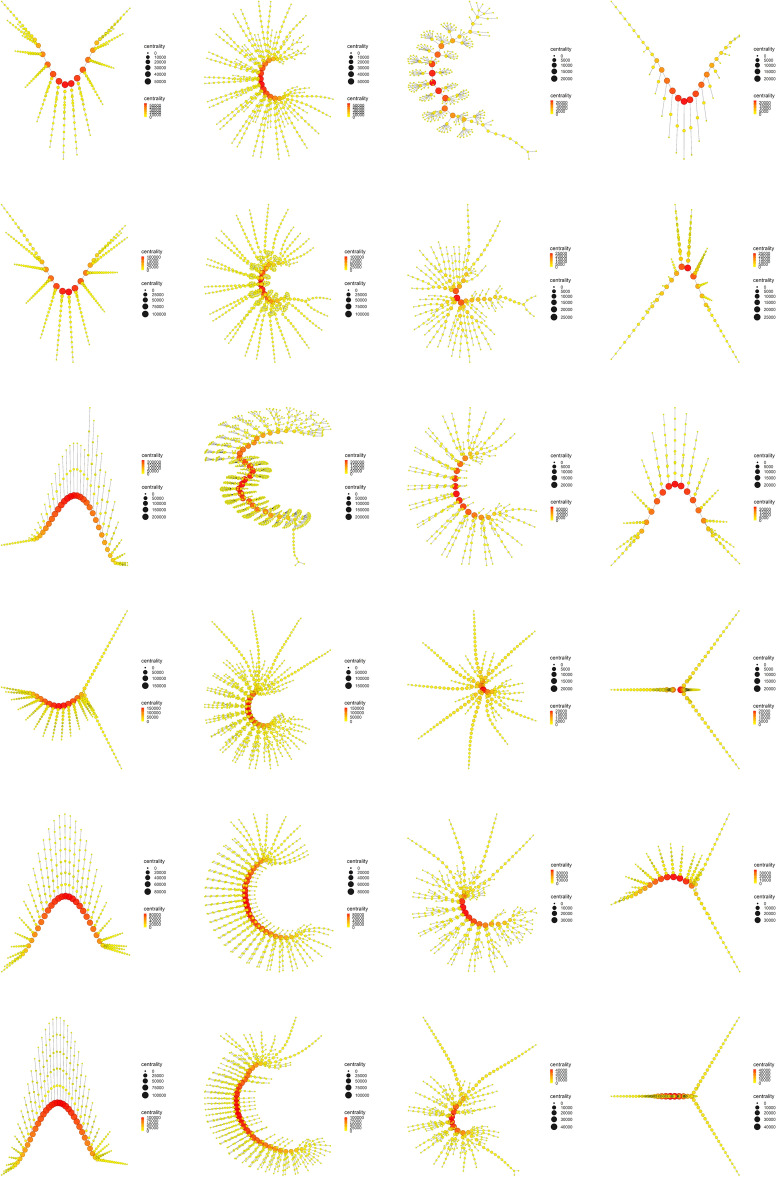
Betweenness centrality. Display and spatialization of arthropod networks’ Betweenness centrality based on two different layout algorithms (Kamada-Kawai (KK) in the two internal columns and MultiDimensional Scaling (MDS) in the two external columns). Arthropod networks are ordered according to the result of the hierarchical clustering. Right branch (column 3 (KK) and 4 (MDS), from row 1 to row 6): *Branchinecta*, *Waptia*, *Olenoides*, *Martinssonia*, *Nebalia*, *Lightiella*. Left branch (column 2 (KK) and 1 (MDS), from row 1 to row 6): *Yohoia*, *Canadaspis*, *Triops*, *Rehbachiella*, *Marrella*, *Speleonectes*. Color (from yellow to red) and size represent the centrality measure value of each node (see inset to the right of each network).

**Figure 11:**
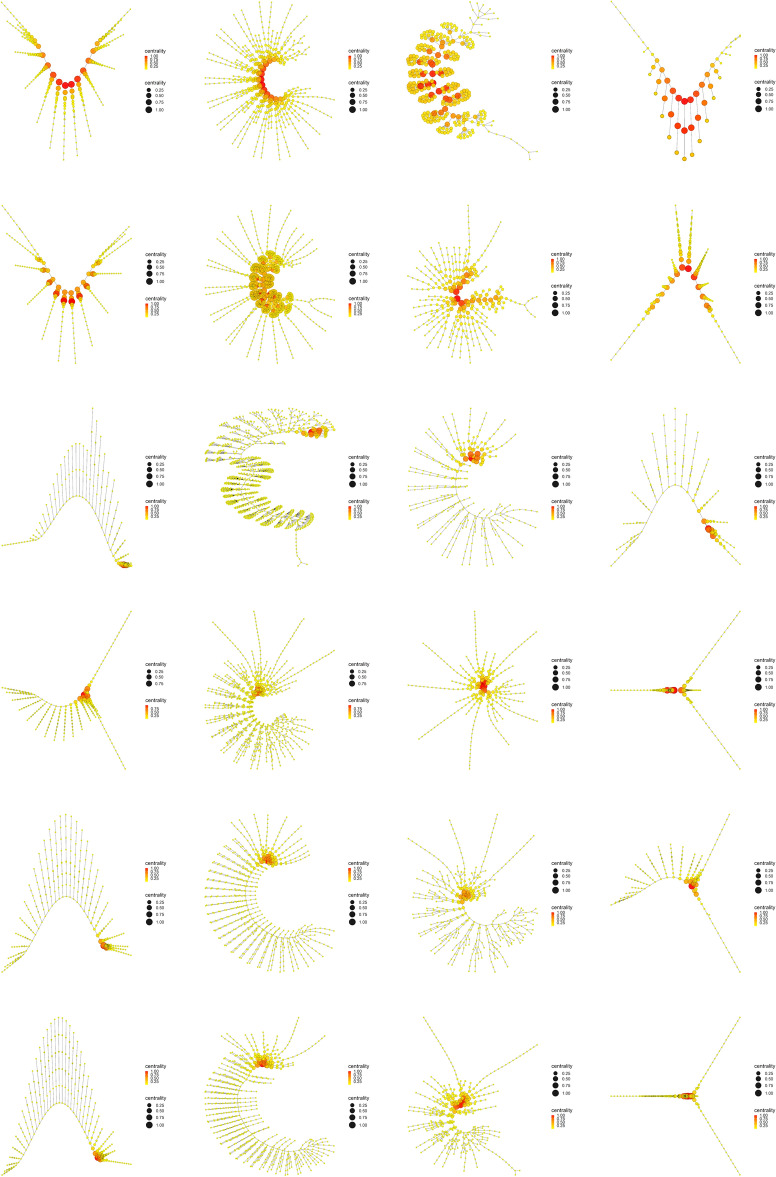
Eigenvector centrality. Display and spatialization of arthropod networks’ Eigenvector centrality based on two different layout algorithms (Kamada-Kawai (KK) in the two internal columns and MultiDimensional Scaling (MDS) in the two external columns). Arthropod networks are ordered according to the result of the hierarchical clustering. Right branch (column 3 (KK) and 4 (MDS), from row 1 to row 6): *Branchinecta*, *Waptia*, *Olenoides*, *Martinssonia*, *Nebalia*, *Lightiella*. Left branch (column 2 (KK) and 1 (MDS), from row 1 to row 6): *Yohoia*, *Canadaspis*, *Triops*, *Rehbachiella*, *Marrella*, *Speleonectes*. Color (from yellow to red) and size represent the centrality measure value of each node (see inset to the right of each network).

The most important evidence of the evolutionary process of primitive arthropods seems to be the decoupling between Betweenness centrality (Fig. 10) and Eigenvector centrality (Fig. 11), or what is the same, the decline or depletion of Eigenvector centrality. While Betweenness centrality remained high and concentrated in the central body axis of all organisms throughout the evolutionary process (red and orange color), both in the left branch (first and second column) and the right branch (third and fourth column), Eigenvector centrality remained high and concentrated in the central body axis of only the most primitive organisms, specifically the two most primitive organisms on the left and right branch (first and second row), that is, *Yohoia* and *Canadaspis*, and *Branchinecta* and *Waptia*, respectively. In later organisms in the evolutionary process, this centrality measure remained high only in the head or cephalic region. This seems to us the most important result of all our work, since it demonstrates that an important property is lost throughout the evolutionary process, a property that seems to allow or facilitate evolution itself (Eigenvector centrality), at the same time that it is increased or intensified a property that seems to slow down and exhaust the evolutionary potential of organisms (Betweenness centrality).

**Figure 12:**
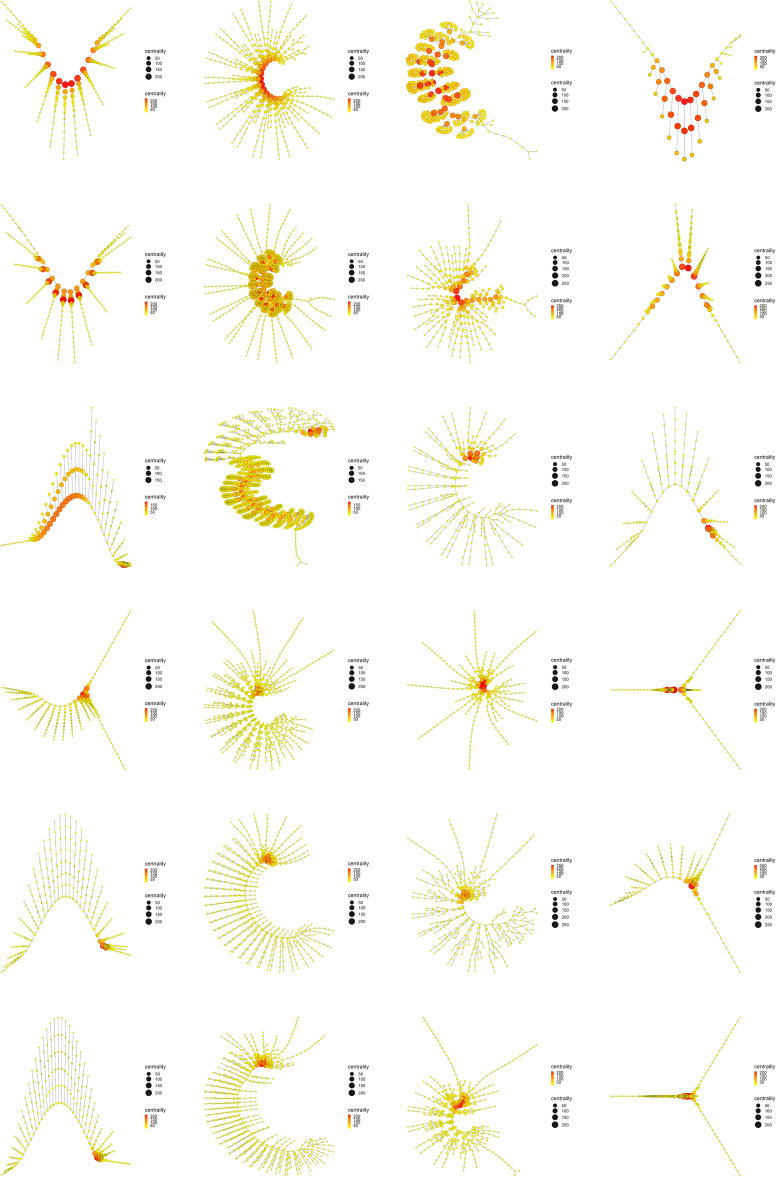
Katz centrality. Display and spatialization of arthropod networks’ Katz centrality based on two different layout algorithms (Kamada-Kawai (KK) in the two internal columns and MultiDimensional Scaling (MDS) in the two external columns). Arthropod networks are ordered according to the result of the hierarchical clustering. Right branch (column 3 (KK) and 4 (MDS), from row 1 to row 6): *Branchinecta*, *Waptia*, *Olenoides*, *Martinssonia*, *Nebalia*, *Lightiella*. Left branch (column 2 (KK) and 1 (MDS), from row 1 to row 6): *Yohoia*, *Canadaspis*, *Triops*, *Rehbachiella*, *Marrella*, *Speleonectes*. Color (from yellow to red) and size represent the centrality measure value of each node (see inset to the right of each network).

Something very similar happened with Katz centrality, a variant of Eigenvector centrality (Fig. 12). The most important difference in this case was that this centrality measure remained relatively high and concentrated in part of the central body axis of *Triops*, specifically in the first 17 abdominal segments that contain appendages (orange color). This can also be verified in Fig. 9. Otherwise, Katz centrality had the same descending behavior throughout the evolutionary process as Eigenvector centrality.

**Figure 13:**
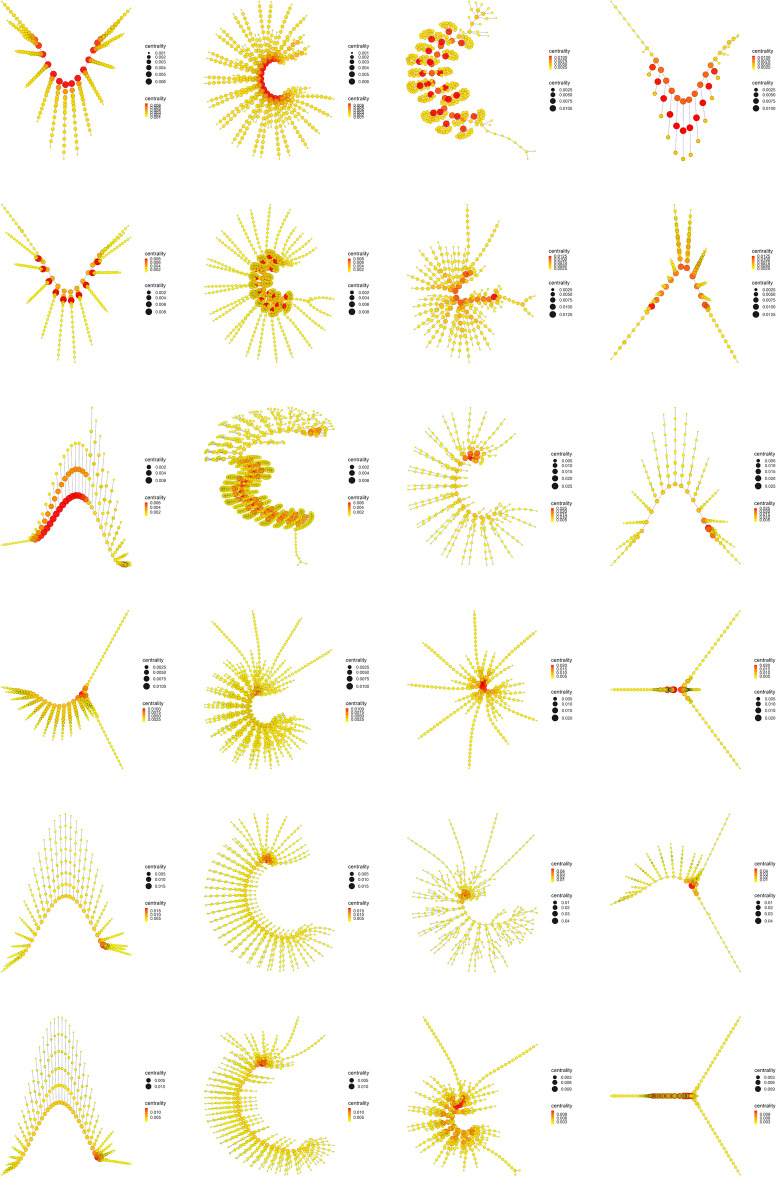
Power centrality. Display and spatialization of arthropod networks’ Power centrality based on two different layout algorithms (Kamada-Kawai (KK) in the two internal columns and MultiDimensional Scaling (MDS) in the two external columns). Arthropod networks are ordered according to the result of the hierarchical clustering. Right branch (column 3 (KK) and 4 (MDS), from row 1 to row 6): *Branchinecta*, *Waptia*, *Olenoides*, *Martinssonia*, *Nebalia*, *Lightiella*. Left branch (column 2 (KK) and 1 (MDS), from row 1 to row 6): *Yohoia*, *Canadaspis*, *Triops*, *Rehbachiella*, *Marrella*, *Speleonectes*. Color (from yellow to red) and size represent the centrality measure value of each node (see inset to the right of each network).

If we now look at what happened with Power centrality (Fig. 13), what we see is a continuation of the trend followed by Eigenvector centrality and Katz centrality. We see that the centrality measure decreases throughout the evolutionary process, but it persists and lasts longer than the previous measures. In the first three groups (rows) of the left branch (*Yohoia*, *Canadaspis* and *Triops*) and the first two groups (rows) of the right branch (*Branchinecta* and *Waptia*), the values were relatively higher (closer to red) than in the two previous cases. This is particularly more noticeable in *Triops*, which obtained intermediate values (orange color) in the body axis in the case of Katz centrality. On the other hand, the Power centrality indices remained at intermediate values in relative terms in the rest of the groups (between dark yellow and light orange), posterior in the two main evolutionary lines. This was particularly noticeable in *Rehbachiella* (fourth group/row of the left branch), and *Martinssonia* and *Lightiella* (fourth and sixth group/row of the right branch), although the effect could also be seen in the rest of the groups.

**Figure 14:**
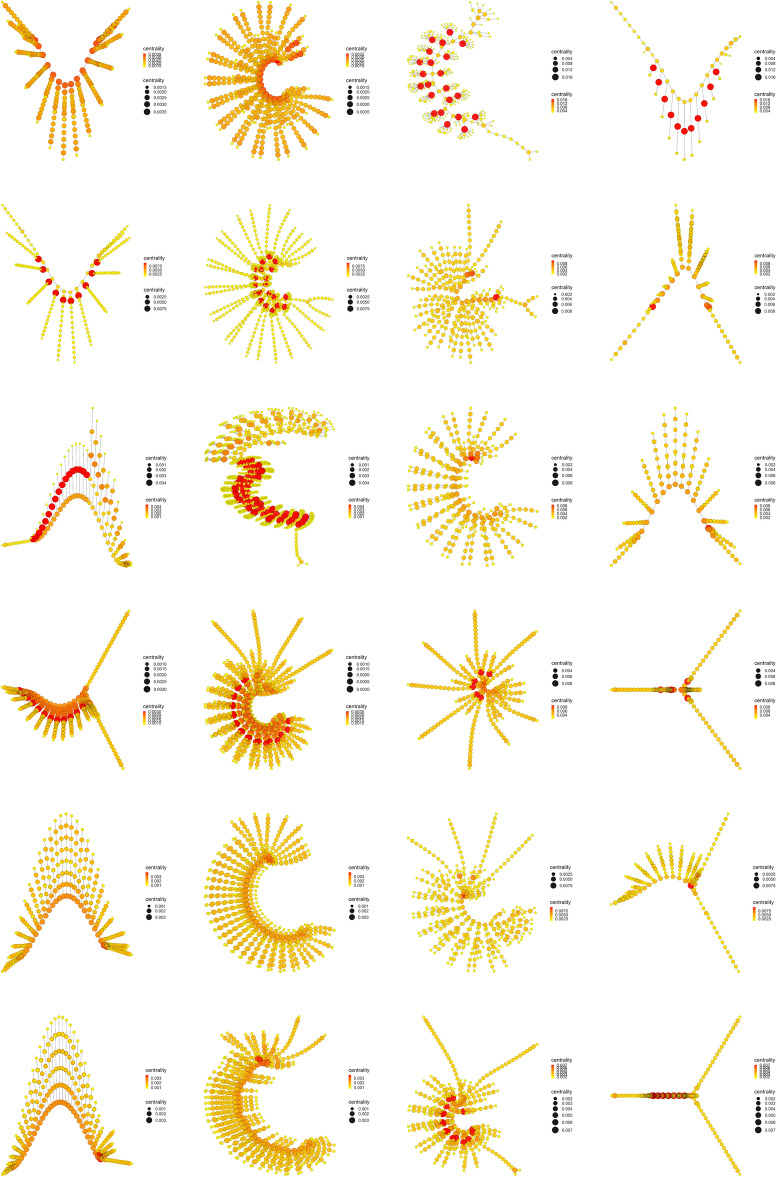
PageRank centrality. Display and spatialization of arthropod networks’ PageRank centrality based on two different layout algorithms (Kamada-Kawai (KK) in the two internal columns and MultiDimensional Scaling (MDS) in the two external columns). Arthropod networks are ordered according to the result of the hierarchical clustering. Right branch (column 3 (KK) and 4 (MDS), from row 1 to row 6): *Branchinecta*, *Waptia*, *Olenoides*, *Martinssonia*, *Nebalia*, *Lightiella*. Left branch (column 2 (KK) and 1 (MDS), from row 1 to row 6): *Yohoia*, *Canadaspis*, *Triops*, *Rehbachiella*, *Marrella*, *Speleonectes*. Color (from yellow to red) and size represent the centrality measure value of each node (see inset to the right of each network).

This trend was further intensified in the last measure derived from Eigenvector centrality, PageRank centrality (Fig. 14). This measure was highly sensitive to nodes with high degree. So much so, that in various cases relatively higher values were obtained in these nodes than in the nodes belonging to the central body axis of the organism. The paradigmatic case of this was *Branchinecta*, in which high values were obtained in the basal nodes of the trunk limbs (red color) and intermediate values in the central body axis (light orange color), a circumstance that had not occurred until now in this organism.

Other interesting results were obtained for Decay centrality (Fig. S1), a measure somewhat related to Closeness centrality. If we look back at Fig. 9, we will see that Closeness centrality, along with Power centrality and PageRank centrality, were the measures that showed a markedly gradual and precise descending bifurcation pattern. Despite this, the Decay centrality pattern did not have these characteristics according to the mean values obtained for these measures. However, visualization of early arthropod networks for this centrality measure shows that a decrease in the measure also occurs throughout the evolutionary process in both evolutionary branches (Fig. S1). In this sense, it was one of the centrality measures that best showed this decrease in centrality throughout both evolutionary branches, and that at the same time lasted and persisted until the end of them, even more than in Power centrality. So much so, that relatively high values were obtained even in the last groups of the left branch (*Rehbachiella*, *Marrella* and *Speleonectes*), detecting a decrease from red to dark orange in the nodes corresponding to the central body axis. Something similar occurred in the right branch, in which not only *Branchinecta* and *Waptia* obtained relatively high values in the central body axis (red color), but also *Olenoides* (light red color), descending in *Martinssonia* and *Nebalia* (orange color), and rising again in *Lightiella* (red color), as also verified in Fig. 9.

Finally, the results obtained for Communicability centrality showed a similarity with those obtained for Power centrality (Fig. S2). Perhaps the small and inconspicuous differences can be found in the two most primitive organisms of both evolutionary branches, in which Communicability centrality obtained relatively slightly higher values in the nodes corresponding to the central body axis. In everything else, both centrality measures showed highly comparable results, which is interesting for studying the relationship that may exist between them.

## Discussion

### Early arthropod evolution as a process of unfolding of a primitive potential complexity

According to our analysis of the evolutionary process of early arthropods used in this work, carrying out a PCA and a HCA, this process is marked by the early bifurcation of two evolutionary branches, which we have called left or large branch and right or small branch. In addition to the presence of *Yohoia* and *Canadaspis* as the most primitive members of the left branch, which are organisms generally considered primitive or basal arthropods, the placement of *Branchinecta* (Branchiopoda: Anostraca) and *Triops* (Branchiopoda: Notostraca) as the first member of the right branch and the third member of the left branch, respectively, was surprising. This places these organisms as playing a more fundamental and originary role than they are generally assigned. On the other hand, organisms that are generally considered very primitive were placed in the last positions of both evolutionary branches. The most paradigmatic case of this was *Marrella*, who ranked as the penultimate representative of the left or large branch.

The evolutionary process as it was ordered and characterized in the present work, revealed that its main characteristic is given by the presence of a descending bifurcation pattern, in which many of the network measures used in this work decreased as they advanced along both evolutionary branches. The clearest and most important results in this regard were found with the majority of the centrality measures (Fig. 9). Centrality measures such as Eigenvector centrality (Fig. 11), Katz centrality (Fig. 12), Power centrality (Fig. 13) and PageRank centrality (Fig. 14), all based on the same fundamental principle, clearly decreased throughout the evolutionary process. Other centrality measures, such as Closeness centrality (Fig. 9), Decay centrality (Fig. S1), related to the previous one, and Communicability centrality (Fig. S2), also decreased. The fundamental difference between them was the speed with which this decline occurred. In this sense, Eigenvector centrality seems to be the earliest detector of primitiveness, since only the first two groups of each evolutionary branch had relatively high values of this measure (red color) in the nodes corresponding to the central body axis: *Yohoia* and *Canadaspis* in the left branch, and *Branchinecta* and *Waptia* in the right branch. For their part, Katz centrality, Power centrality and PageRank centrality (in that order) lasted and persisted more and more throughout the evolutionary process, the latter being very sensitive to nodes with high degree. In this sense, Decay centrality was the measure that best showed this behavior, being able to detect relatively high values (dark orange, or even light red) even in the last groups of both evolutionary branches. All this demonstrates the robustness of the results obtained, especially the structure of the evolutionary tree developed, and reveals an underlying logic in this evolutionary process.

Eigenvector centrality is a centrality measure that measures the influence of a node within the network^50^. Based on the concept that connections to high-scoring nodes contribute more to a node’s centrality than connections to low-scoring nodes, this measure assigns relative scores to all nodes in the network. A high score means that a node is connected to many nodes, which in turn are connected to many nodes. In this manner, this centrality measure does not measure the quantity but the quality of the connections. Another way of looking at it is that Eigenvector centrality is based on the value of the neighbors of a certain entity or node, and not on the intrinsic value of the entity or node itself. This measure is based on the eigenvalue, which means that the value of a node is based on the value of the nodes connected to it: the higher the second, the higher the first. All this leads us to the interpretation that a node with a high Eigenvector centrality is connected to prominent, popular, important nodes, and even if it itself does not have the same importance, it can take advantage of the popularity and influence of its connections. In this sense, a node with a high Eigenvector centrality is a node with many influential ties. Now, how can this interpretation of social networks be translated to the case of a network that represents a morphological structure? What does this *influence* and *popularity* represent in morphological terms? The concept of influence is a concept that can have a translation in terms of morphological evolution and development. Influence somehow represents the power or capacity to control and alter the development of something or someone. In other words, influence represents the power that something or someone has to cause changes in others. A node with high influence, i.e. a node with high Eigenvector centrality, in a network that represents a morphological structure, then represents a node with the power or capacity to cause morphological changes in other nodes, that is, a node that can control and alter the evolutionary development of other nodes. A concept that in this work we are going to call *evolutionary developmental potential*. This concept has a concrete and comparable correlate in developmental biology. There is a concept in this field that is generally called developmental potential, which describes the potential or capacity that a cell or groups of cells have to generate and produce different cell types in themselves and in their neighbors. This potential is reduced as the development of the organism progresses. In our case, we apply it to the evolutionary field, although we do not consider it independent and separate from development (hence its name), and we are not applying it to the case of cells or groups of cells, but to morphological units or structures.

What conclusions can we then draw from this new concept of evolutionary developmental potential represented and quantified by Eigenvector centrality? The results showed that Eigenvector centrality is higher in the most primitive arthropods and that it is drastically reduced throughout the evolutionary process, both in quantitative terms (normalized mean values, Fig. 9) and qualitative terms (relative distribution of the centrality measure within each group, Fig. 11). In the most primitive groups (*Yohoia* and *Canadaspis* in the left branch, and *Branchinecta* and *Waptia* in the right branch), the centrality measure was located preferentially in the nodes corresponding to the central body axis, although it was also located in nodes of the limbs or appendages. Let’s investigate this distribution in more detail. In *Yohoia*, Eigenvector centrality values were relatively high (orange to red) in the nodes corresponding to the body axis segments of the head and trunk, except for the segment corresponding to the eyes (14 segments); and to a lesser extent in the first article of their corresponding appendages, except for the two most distal, that is, the great appendage and the trunk appendage 10 (12 pairs of cephalic and trunk appendage basipods). In *Branchinecta*, Eigenvector centrality values were relatively high (orange to red) in the nodes corresponding to the body axis segments of the trunk (11 segments) and in the nodes corresponding to the base of the trunk limbs (11 limb base pairs). In *Canadaspis*, Eigenvector centrality values were relatively high (orange to red) in the nodes corresponding to the body axis segments of the trunk and the first and second maxilla (10 segments), and in the first article and the outer ramus lobe of their corresponding appendages (10 pairs of basal articles and 10 pairs of outer ramus lobes). The highest values were found in the outer ramus lobes. Finally, in *Waptia*, Eigenvector centrality values were relatively high (orange to red) in the nodes corresponding to the body axis segments of the cephalothorax and post-cephalothorax (13 segments), and we could also include the first article (podomere) of the post-maxillular appendages 1 to 3 (3 pairs of proximal podomeres). On the other hand, in the rest of the evolutionary series, Eigenvector centrality was only detected and concentrated in the cephalic region of the organisms. Thus, for example, in *Triops*, the highest value of Eigenvector centrality was found in the carapace (red color), followed by all the nodes to which it was connected: all the cephalic segments (5 segments), including the two eyes (orange color). Something similar occurred in *Rehbachiella*, in which the highest value of Eigenvector centrality was found in the cephalic shield (red color) and, then, the nodes to which it was connected: all the cephalic segments (6 segments, orange color). In general terms, this pattern was repeated in all other organisms: Eigenvector centrality was high first in the cephalic/head shield (red color), and then in the cephalic segments to which it was connected (orange color). In this manner, we can affirm that the evolutionary developmental potential declines throughout the evolutionary process, going from being located along the entire organism body axis, preferably the segments of the head and thorax, to being located only in the cephalic region, cephalic/head shield and connected cephalic segments. This means that primitive organisms have a much more extensive capacity for evolutionary-developmental change, and that they have the potential to generate morphological changes along almost their entire body axis (head and thorax). Their cephalic and thoracic segments, and the most proximal articles of their appendages, have the capacity to cause morphological changes in themselves and their neighbors. It is in this sense that these nodes/segments have *influence* on their surroundings.

We now turn to analyze Katz centrality^51^, a measure related to Eigenvector centrality. The main difference between this centrality measure and the previous one is that with this measure *Triops* obtained high values in its central body axis (Fig. 12). Relatively high values of Katz centrality (orange to red color) were observed not only in the cephalic region (carapace, associated cephalic segments and eyes), as occurred with Eigenvector centrality, but also in the body segments corresponding to the abdominal segments with appendages, that is, the first 17 abdominal segments. We could also include the protopods or basal articles of the 17 pairs of abdominal appendages. The rest of the organisms gave a result almost identical to that obtained with Eigenvector centrality. What is the reason for this difference between Eigenvector centrality and Katz centrality? Katz centrality is a centrality measure that computes the relative influence of a node by measuring its distance to all other nodes in the network, penalizing each path or connection by an attenuation factor (alpha parameter)^51^. Depending on the value of this parameter, this measure can range from Degree centrality (when alpha approaches 0) to Eigenvector centrality (when alpha approaches the inverse of the largest eigenvalue). This can be interpreted as Degree centrality measuring the *local influence* of a node, while Eigenvector centrality measures the *global influence* of a node. In this manner, Katz centrality is a centrality measure that measures both the local and global influence of a node^62^. Following this line of reasoning then, the novelties found with Katz centrality are due to the fact that this measure is detecting nodes with more local and circumscribed influences than Eigenvector centrality. This means that the evolutionary developmental potential present in the abdominal segments of *Triops* is important, but has a more limited influence than that of the cephalic segments. Its circle and area of influence to cause morphological changes is shorter and less far-reaching.

If we now study in detail Power centrality^53^, a centrality measure derived from Eigenvector centrality, we see that this trend of higher detection *sensitivity* increases even further. With this measure, the values generally increase in all organisms (Fig. 13). In *Triops*, for example, the values obtained for the first 17 abdominal segments are now relatively higher (red color). On the other hand, the already relatively high values in primitive organisms, especially in their central body axes, are further intensified and increased, the most notorious case being the bases of *Branchinecta*’s trunk limbs. However, the most important difference is that organisms that are posterior in the evolutionary process now obtain relatively higher values for this measure. This occurs mainly in *Olenoides*, *Rehbachiella*, *Martinssonia* and *Lightiella*. In *Olenoides*, the segments of the thorax and pygidium (12 segments) now appear orange. In *Rehbachiella*, the 11 segments of the thorax also appear orange. In *Martinssonia*, the same happens with the rest of the cephalic and thoracic segments with appendages (3 segments), and even with the coxa and the base of appendages 2, 3 and 4. For its part, in *Lightiella*, the thoracic segments with well-developed appendages (first 7 thoracic segments), and the protopods of the maxillae and thoracopods 1 to 7 (all of 2 articles, except the last one of only 1 article), now appear orange. These results lead us to ask what Power centrality measures and what *power* means in this context.

Both Eigenvector centrality^50^ and Power centrality^53^ were developed by Phillip Bonacich. Eigenvector centrality was developed as a centrality measure in which the centrality of a unit consisted of its summed connections to others, weighted by their respective centralities. Power centrality was developed to allow greater flexibility. An extra parameter, attenuation factor or beta parameter, allows to vary the degree of dependency of the score of a unit with respect to the score of the other units. In general terms, this attenuation factor can be interpreted as the probability that a communication or information from one node is transmitted to its neighboring nodes. The magnitude of the beta parameter then reflects the degree to which the communication is transmitted locally or globally. Low values of this parameter predominantly focus and evaluate the local structure, while high values evaluate the position of a node in the structure as a whole. In this sense, the beta parameter can be seen as a radius of influence within which one wants to evaluate the centrality of a node^53^. In this manner, as we used a low value of beta parameter (0.2), we could define that Power centrality is measuring the local evolutionary developmental potential, that is, the power of influence of a morphological unit to cause changes in its immediate surroundings and vicinity. It makes sense then that this centrality measure detects zones of influence not detected by Eigenvector centrality and Katz centrality. Therefore, we could define the following scenario: Eigenvector centrality would be revealing long-range zones of influence, Katz centrality would be revealing medium-range zones of influence, and Power centrality would be revealing short-range zones of influence.

These results are highly consistent and point to a scenario in which the evolutionary developmental potential gradually decreases in magnitude and scope throughout the evolutionary process. This means for us the first empirical evidence of our theory of evolution as a process of unfolding^14^. In a previous work, we had found evidence of the theory in the case of ontogenetic development^15^. In this case, we can affirm, assuming the plausibility of the evolutionary tree developed by HCA, that the evolutionary process of early arthropods is largely marked by the decline and depletion of the evolutionary developmental potential, measured mainly by Eigenvector centrality, Katz centrality and Power centrality. This potential unfolds and actualizes in a greater extensive complexity of the organisms, measured by a large part of the topological descriptors (Groups 1 and 3), but also by various network parameters (Groups 1 and 3) and some centrality measures (e.g. Betweenness centrality).

An interesting question is whether this evolutionary developmental potential coincides with what we have called intensive complexity in a previous work^15^. It is not a simple question, but we can draw some conclusions based on the results obtained so far. In the previous work, intensive complexity was represented by complexity measures, while extensive complexity was represented by topological descriptors. In this work, the dimension in which these network measures move is mainly given by dimension 1 of the PCA (Fig. 3): topological descriptors are concentrated on the left of the dimensional space (PCA1 negative), and complexity measures are located on the right (PCA1 positive). However, as we have already seen, the temporality of the evolutionary process seems to be given in this analysis predominantly by dimension 3: the most primitive organisms are located in the upper region of the dimensional space (PCA3 positive, Fig. 2). This seems to be indicating that the process that we are revealing in this work, which allowed us to define and create the so-called evolutionary developmental potential, introduces a new dimension to the evolutionary developmental process. In this manner, apparently the developmental process and the evolutionary process involve different variables and dimensions, although both would imply a process of unfolding. This evidently should be further studied in future work.

### What is it like to be primitive?

An important question that arises after the results obtained in this work is what it means for an organism to be primitive. More precisely, what morpho-topological structure characterizes a primitive organism? Throughout the history of the study of arthropod evolution, the idea that the archetypal primitive arthropod, the *Urarthropod*, was characterized by a simple, monotonous and repetitive structure has been considered generally accepted. Thus, for example, Hessler & Newman (1975)^9^, choosing Cephalocarida (*Hutchinsoniella*), Leptostraca (*Nebalia*) and Notostraca (*Lepidurus*) as representatives of primitive crustaceans, depicted the morphological structure of their urcrustacean as that of an organism consisting of a head and a thorax made up of a repetitive series of about 22 segments, each composed of a pair of 7-article stenopodous limbs. All appendages of this urcrustacean (mandibles, maxillula 1, maxillula 2 and thoracic limbs) were equal and uniform. One cannot help but see in this reconstruction of the hypothetical urcrustacean, despite all the differences that can be found, the supposedly primitive crustacean that would be discovered a few years later, in 1981, which would be called *Speleonectes*, and which would be placed in a new class called Remipedia. At that time, the authors considered that their reconstruction of the hypothetical primitive crustacean had great similarity and resemblance to trilobites, which is why they proposed *A trilobitomorph origin for the Crustacea*. Thus, for these authors, the primitive representative of arthropods in general and crustaceans in particular consisted of an organism with a high degree of serial homology and stenopodous linear limbs. This is the classic and standard view of a primitive arthropod or crustacean. Our work provides clear and compelling evidence against this classical view. All organisms that had a morpho-topological structure similar to that of this classical view were considered late organisms in the evolutionary process, and organisms in which the evolutionary developmental potential had already decreased substantially, that is, their potential for change had been reduced, constrained, and therefore structurally and topologically localized. On the other hand, organisms that were characterized as primitive had a different morpho-topological structure. They could present a high degree of serial homology, but unlike the classical or standard model, these organisms presented a much more complex segmental topological structure. The segments of these organisms consisted of true *irradiation centers* from the central body segment: a *plexus*. This word is very significant in our context. A plexus is an interwoven network of parts or elements in a structure or system. This expresses very well the type of structure that we are trying to describe. But there are more interesting things in this concept. If one traces the origin of this concept, one finds that a plexus is a structure that is *folded*. We could find here the reason why this structure, the plexus, represents a morpho-topological structure of concentrated complexity, with the capacity to unfold in a diversity of forms thanks to the influential potential that it intrinsically possesses.

There are bibliographic precedents that follow the same line of thought developed in this work. Olesen et al.^63^ proposed that segmented trunk limbs have evolved from phyllopodous limbs, based on the study of the embryological development of two branchiopods, one with phyllopodous limbs (*Cyclestheria*) and the other with stenopodous limbs (*Leptodora*). The empirical and logical basis for reaching this conclusion was that both organisms began the development of their limbs in a similar way: “In both species the limbs are formed as ventrally placed, elongate, subdivided limb buds”^63^. This work clearly shows that, during its embryological development, *Leptodora* goes from having phyllopodous limbs to having stenopodous limbs. The evolutionary translation of this developmental result has the character of inferential, but in any case it is well grounded and justified in the relative phylogenetic positions of both groups. Borradaile^64^ had already speculated about the possibility that the primitive crustacean limb was not stenopodous but phyllopodous, and that articles of stenopodous limbs originated from the endites of phyllopodous limbs. After him, Fryer^65^ proposed a similar hypothesis, pointing out that primitive arthropod appendages did not have stenopodous limbs. The additional contribution of our work regarding this specific point is to provide a theoretical and rational hypothesis about the reason and cause of this evolutionary change: the segmental morpho-topological structure of organisms that have phyllopodous limbs is a structure that contains within itself a higher evolutionary developmental potential, which allows it to have the intrinsic capacity to produce morphological changes in its structure and in its more or less close environment.

### Wonderful potential life: early arthropod evolution and the nature of history

In his book *Wonderful Life*, Stephen Gould^66^ makes a revision of Burgess Shale fossils, which date back about 508 million years, with the idea of correcting and replacing the iconography of evolution as “cone of increasing diversity”, which he personifies in the figure of Charles Walcott, for the iconography of “decimation and diversification”, which he tries to personify in the figure of Harry Whittington. More profoundly, however, Stephen Gould not only pointed against the usual iconography of evolution as a cone or tree of increasing diversity, but also against the view of history as a progressive process of increasing complexity, to propose a model based on *contingency*: “the “pageant” of evolution as a staggeringly improbable series of events, sensible enough in retrospect and subject to rigorous explanation, but utterly unpredictable and quite unrepeatable”^66^, p. 14. Gould posits historical contingency as a third way, a middle way, to the known extremes of historical determinism and complete randomness.

According to Gould, the iconography of the cone made Charles Walcott’s interpretation of Burgess Shale organisms inevitable. These animals were found at the narrow base of the cone, at the origin of pluricellular life, so their diversity must be limited to a basic morphological simplicity. In this manner, these organisms had to be considered as primitive forms of modern groups, that is, as ancestral forms that, thanks to a temporal increase in complexity, progressed to some modern form. Consequently, Walcott interpreted Burgess Shale organisms as primitive members of known modern arthropod groups. Also according to Gould, the reconstructions of Burgess Shale organisms by Harry Whittington and his colleagues challenged the iconography of the cone and “turned the traditional interpretation on its head”^66^, p. 47. Unlike Walcott, Whittington interpreted Burgess Shale organisms as new morphological groups, not belonging to any modern group. Whittington would then have inverted the cone of life: “The sweep of anatomical variety reached a maximum right after the initial diversification of multicellular animals. The later history of life proceeded by elimination, not expansion. The current earth may hold more species than ever before, but most are iterations upon a few basic anatomical designs”^66^, p. 47. According to this view then, most or all *body building plans*, i.e. *Bauplan*, of arthropods appeared at the beginning and not at the end of the evolutionary process: “The maximum range of anatomical possibilities arises with the first rush of diversification”^66^, p. 47. We could rewrite this in this other way: “The greatest *evolutionary developmental potential* is found at the beginning of the evolutionary process”. This is what we found in our work. However, we did not detect an inversion of the cone of life. This may be due to the clustering method used to build the evolutionary tree, which somehow presupposes an arborescent structure. Even so, if we bring together all the results obtained in this work, from the PCA to the Centrality measures, we can see a clear rationality, directionality and sense of the evolutionary process, which allows us to think that the results found and organized in the evolutionary tree are very well supported by empirical evidence.

This means that at the beginning of the evolutionary process of arthropods there was the greatest potential for the generation of new body building plans, that is, that primitive arthropods had a greater capacity to generate new forms, and more unique and different forms, than modern arthropods. Here we must also take into account that some of these primitive arthropods with greater potential for change are organisms that still exist today, according to the results of our work. However, the fact that these primitive arthropods have a greater potential does not mean that they are more complex. On the contrary, they can be quite simple. The problem is what one understands by complex and simple. This question is rather complex than simple. According to the results of this work, we could say that the simple has a greater potential to generate new forms. However, not any simple form has a high potential. We have already seen what types of simple structures have a high potential: those that can have serial homology, but their segments have a plexus morpho-topology. On the other hand, very complex structures may have a very low potential for change. Their structural elements are so committed, interconnected and integrated into the structure as a whole, that it is very difficult for these structures to change and evolve. This is compatible with Rupert Riedl’s concept of *burden*^67^.

Returning to Gould, he considers that even the inverted iconography he proposes for the early history of arthropods, based on the Burgess Shale findings, can still be interpreted in the “traditional” terms of evolutionary predictability and directionality: “We can abandon the cone, and accept the inverted iconography, yet still maintain full allegiance to tradition if we adopt the following interpretation: all but a small percentage of Burgess possibilities succumbed, but the losers were chaff, and predictably doomed. Survivors won for cause—and cause includes a crucial edge in anatomical complexity and competitive ability”^66^, p. 48. For Gould, the inverted iconography enables a different alternative that for him was prevented by the iconography of the cone: that survivors did not survive due to a justified cause, such as greater morphological complexity, but simply due to mere accidents, such as unpredictable environmental catastrophes. It is in this context that Gould proposes the “experiment”, rather the metaphor, of “replaying life’s tape”: “You press the rewind button and, making sure you thoroughly erase everything that actually happened, go back to any time and place in the past—say, to the seas of the Burgess Shale. Then let the tape run again and see if the repetition looks at all like the original. If each replay strongly resembles life’s actual pathway, then we must conclude that what really happened pretty much had to occur. But suppose that the experimental versions all yield sensible results strikingly different from the actual history of life”^66^, p. 48. In the latter case, he would verify his hypothesis of historical contingency. This interpretation of Gould has its drawbacks. In the first place, the metaphor of “replaying life’s tape” is confusing, and perhaps the appropriate metaphor would have been that of “time travel”. If life is a tape, then each time we rewind it we will see the reproduction of the same history of life. Now, this metaphor raises various questions: would the contingencies and accidental events to which Gould alludes and resorts be *internal modifications in the content* of the tape or rather *external alterations to the structure* of the tape? In other words, would “impacts of extraterrestrial bodies” produce modifications to the content of the tape or its structure as a tape itself? For Gould, the answer seems to be the first option: “any replay of the tape would lead evolution down a pathway radically different from the road actually taken”^66^, p. 51. However, it is clear that Gould adopts an externalist view of life and history in this text, and such a view can only cause external alterations to the tape’s structure, and is incapable of generating internal modifications to the tape’s content. The truth is that the iconography, whether in an increasing or inverted cone, does not change the “view of life” in the sense that he believes: one can have a contingent conception of life with both iconographies. The problem is that in both cases Gould assumes that the shape of the cone modifies and alters the evolutionary process, as if it were a mold to which the evolutionary process must fit. Our work brings a new vision to life evolution, which is even more faithful to the idea of “life’s tape”. Our proposal is that life has a potential that unfolds and actualizes in what we call history. The potentiality of life would be the tape’s content, and its reproduction would be history itself. The results of our work provide evidence that life evolution has sense and directionality, that is, that it follows a path marked by intrinsic internal patterns, which can be studied and revealed, and which could even be predicted.

### A theory of evolution as a process of unfolding and the future of evolutionary theory

We have recently published an alternative evolutionary theory based on the concept of *unfolding*^14^. This concept is the original meaning of the word evolution, which comes from the Latin word *evolutio*^68^. This theory is based on four concatenated logical principles. The first principle establishes that *the more complex cannot be generated by the simpler*. The logical foundation of this principle is that something simpler does not have the necessary *in-formation* for the formation of something more complex. The second principle establishes that, as a consequence of the first principle, there must be a *maximum complexity* that ensures and guarantees the formation of the less complex. This principle in turn guarantees that there is no infinite regress. The third principle establishes that, as a consequence of the previous principles, there must exist an *ideological matrix* consisting of a *morphogenetic field* formed by the successive temporal stages-folds of the evolutionary process. The unfolding of this morphogenetic field generates the evolutionary history from the simpler to the more complex. The fourth principle establishes that, as a consequence of the previous principles, the evolutionary process is in itself a process of actualization and projection of *virtual potentialities*, a process that is driven by the action of *teleological-purposeful formal agents*. This principle also establishes a dualism between the *virtual* and *pre-existing* ideological matrix, and the actual and real evolutionary process that we see happening historically and temporally.

This theory has a number of important characteristics and consequences. First, it is a *preformationist* theory: each stage of the evolutionary process represents the unfolding and actualization of a preformed morphogenetic field. Second, it is a *teleological* theory: each event of the evolutionary process represents the actualization and projection of a potentiality by a teleological-purposeful formal agent. Third, it is a theory based and sustained on the concept of *consciousness*: each of these processes of actualization represents a process of consciousness expansion on the part of these agents. What these agents make conscious is the information contained in the form of morphogenetic fields in the ideological matrix.

This theory, despite being very different from current evolutionary theory, has several advantages and virtues. Being based on logical principles, the theory is falsifiable and empirically verifiable. This is basically what we did in this work and in a previous work^15^. On the other hand, the concept of natural selection presents its difficulties and limitations in this area, and has serious logical inconsistencies^8,14,69^. The current theory also presents difficulties in explaining how random changes and mechanical activities at the genetic level are translated into a form, and into an organism capable of purposeful and agentic behavior. Generally speaking, it has difficulty explaining how lower levels of biological organization generate higher levels of biological organization. The theory we propose has the potential to explain this and other properties of living organisms, such as the emergence of life, the increase of complexity in organisms and the existence of purposefulness in nature. In other words, this theory has the potential to explain how from organisms as simple as bacteria and protozoa, organisms as complex as human beings were generated, and how it is possible that from a single cell an organism capable of purposeful behavior and conscious thinking can be generated^69^.

In recent years, attempts have been made to integrate some of these concepts into evolutionary theory. Thus, for example, a book has recently been published that attempts to integrate the concepts of *teleology-purposefulness* and *agency* into evolutionary theory^70^. The editors, among whom are renowned researchers such as Peter Corning, Stuart Kauffman and Denis Noble, prefer the use of the term *teleonomy* to talk about the first of these concepts. The concept of teleonomy was developed by Colin Pittendrigh to differentiate it from the Aristotelian concept of teleology^71^. Pittendrigh’s intention was to get rid of the final cause, the *télos*, and maintain the existence of an “end-directed mechanism”, carried out by “end-directed systems”. Some authors consider the introduction of this term useful^72^, while others consider it innocuous or unproductive^73^. For us, the change is not trivial: it represents the elimination of agency, and the reduction of a living being to a physical system with negative feedback loops. We have already dealt with the problem of the so-called “organicism” in another work, and its intimate relationship with Bertalanffy’s systems theory and cybernetics^74^. In any case, we do not believe that all the authors of the book think that way, since among them is Denis Walsh, who has written extensively on teleology and agency^75,76^. We have written at some length about these topics^74,77–79^, in addition to being concepts integrated into our theory described above^14^.

On the other hand, and even more related to the present work, Jaroslav Flegr has developed a theory of frozen evolution^80^ and, in collaboration, has recently postulated the existence of a *macroevolutionary potential*^81^. According to these authors, this macroevolutionary potential measures the capacity of variation of a given species, that is, the probability of producing major evolutionary innovations. This potential decreases throughout evolution, which eventually leads to a frozen state. As can be seen, this potential is similar to the *evolutionary developmental potential* that we are proposing in this work, but from an essentially different conceptual framework. The concept developed by these authors basically occurs within the framework of a selective process of generation of new evolutionary lineages, both at the genotypic and phenotypic levels. The authors explain this process of depletion of macroevolutionary potential and evolutionary freezing, largely resorting to the dynamics of genetic and phenotypic modules. As these modules become more and more integrated and form more and more interconnections, their ability to change is drastically reduced. This is basically what Rupert Riedl explains with his concept of burden: as morphological structures acquire greater connections, they also acquire greater internal commitments, and that structure is transformed into a more internal and deeper component of the system^67^. There are numerous theoretical and empirical studies that support the decline of an evolutionary potential^66,67,82–85^. In general terms, conceptual frameworks that attempt to explain the increase in complexity through the emergence of new and higher hierarchical levels, such as the works of Daniel McShea^86–88^, are highly compatible with the vision proposed in this work and in our theory. Recently, this author, alone and in collaboration, has also attempted to integrate and make compatible the action of hierarchical morphogenetic fields with the presence of teleological behavior or activity^89,90^, a topic that we have initially addressed in another work^74^, and that we have included in our theory^14^.

Finally, our conceptual and theoretical framework is aligned with an entire idealist morphological tradition that proposes the existence of a building plan, a *Bauplan*, that controls and directs biological formation and development, a *Bauplan* that we have reformulated in the terms of a morphogenetic field. This tradition can be traced back to Goethe himself, with his concept of *Urbild*, “an anatomical archetype [...] a general picture containing the forms of all animals as potential”^91^. This tradition was later continued by Geoffroy Saint-Hilaire, with his theory of analogues and his principle of connections^92^, and Richard Owen^93^. More contemporarily, this line of thought has been continued by authors such as D’Arcy Thompson^94^, Stephen Gould^95,96^, Rupert Riedl^67^ and Brian Goodwin^97^. This line of thought ultimately maintains that there must be a plan, blueprint or prototype that gives sense, directionality, and directs the morphogenetic process in evolution and development. Therefore, this process does not occur randomly, nor is any form possible, that is, the plasticity promoted by the adaptationist program of mutation and adaptation does not exist^98^. This is what our theory supports, and this work provides empirical evidence in favor of it. The future will tell what will be the fate of this theory, and of all the new concepts and ideas it proposes.

### Supplementary Information

Supplementary Information.

Supplementary Figure S1.

Supplementary Figure S2.

## Data Availability

All data necessary to support the conclusions of this article are provided as Supplementary Information.
