# Supplementary material for: A network analysis of early arthropod evolution and the potential of the primitive
Source: Sci Rep. 2024 Jan 4;14:503. doi: 10.1038/s41598-023-51019-x (PMC10766614; doi:10.1038/s41598-023-51019-x)
Supplement: Supplementary file 2 — Supplementary Figure S1. [file 41598_2023_51019_MOESM2_ESM.zip › Figure S1 (legend).docx]

# Figure S1

**Decay centrality.** Display and spatialization of arthropod networks' Decay centrality based on two different layout algorithms (Kamada-Kawai (KK) in the two internal columns and MultiDimensional Scaling (MDS) in the two external columns). Arthropod networks are ordered according to the result of the hierarchical clustering. Right branch (column 3 (KK) and 4 (MDS), from row 1 to row 6): *Branchinecta*, *Waptia*, *Olenoides*, *Martinssonia*, *Nebalia*, *Lightiella*. Left branch (column 2 (KK) and 1 (MDS), from row 1 to row 6): *Yohoia*, *Canadaspis*, *Triops*, *Rehbachiella*, *Marrella*, *Speleonectes*. Color (from yellow to red) and size represent the centrality measure value of each node (see inset to the right of each network).
