# Supplementary figures and images for: A network analysis of early arthropod evolution and the potential of the primitive
Source: Sci Rep. 2024 Jan 4;14:503. doi: 10.1038/s41598-023-51019-x (PMC10766614; doi:10.1038/s41598-023-51019-x)

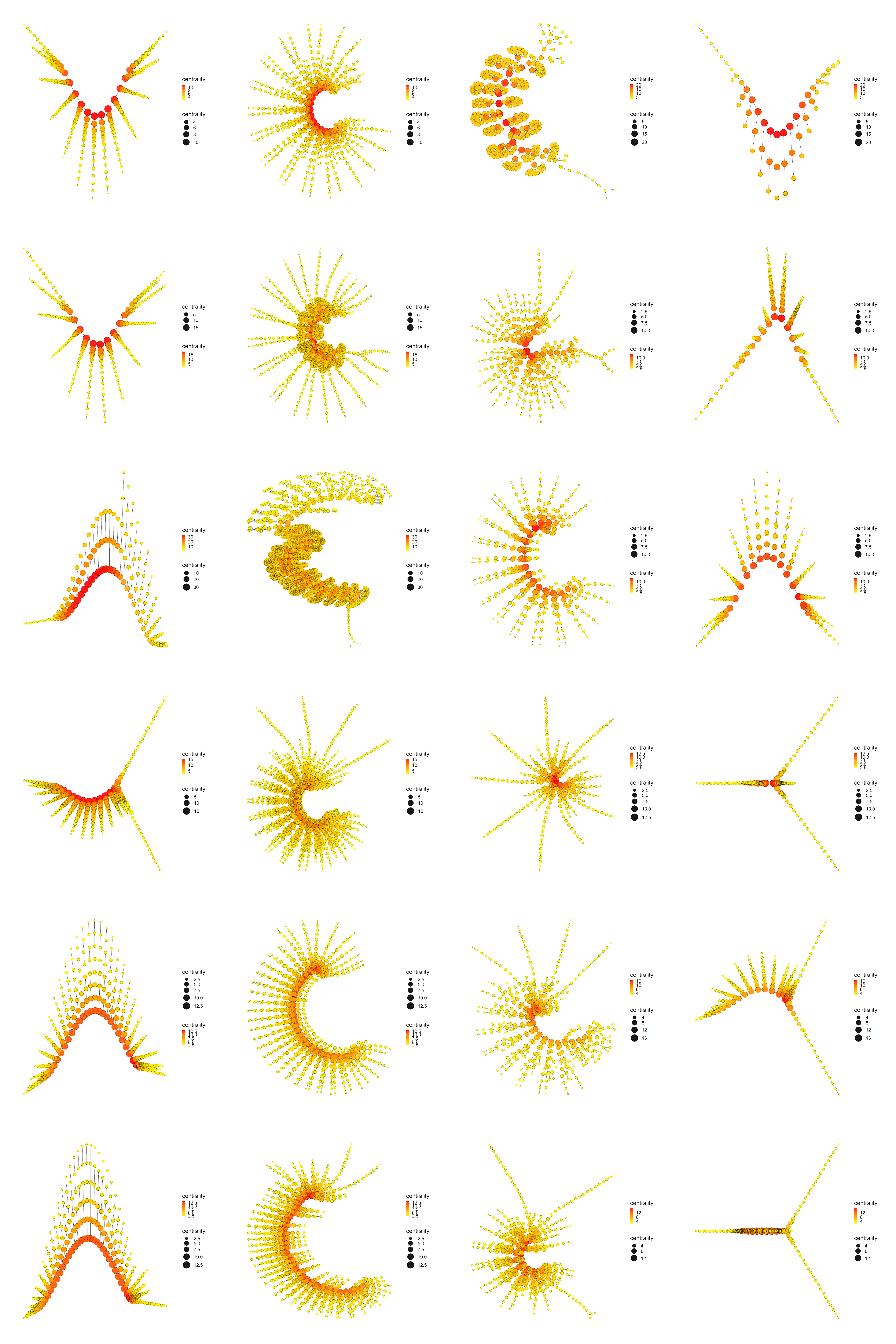

Supplement: Supplementary file 2 — Supplementary Figure S1. [file 41598_2023_51019_MOESM2_ESM.zip › Figure S1.tif]

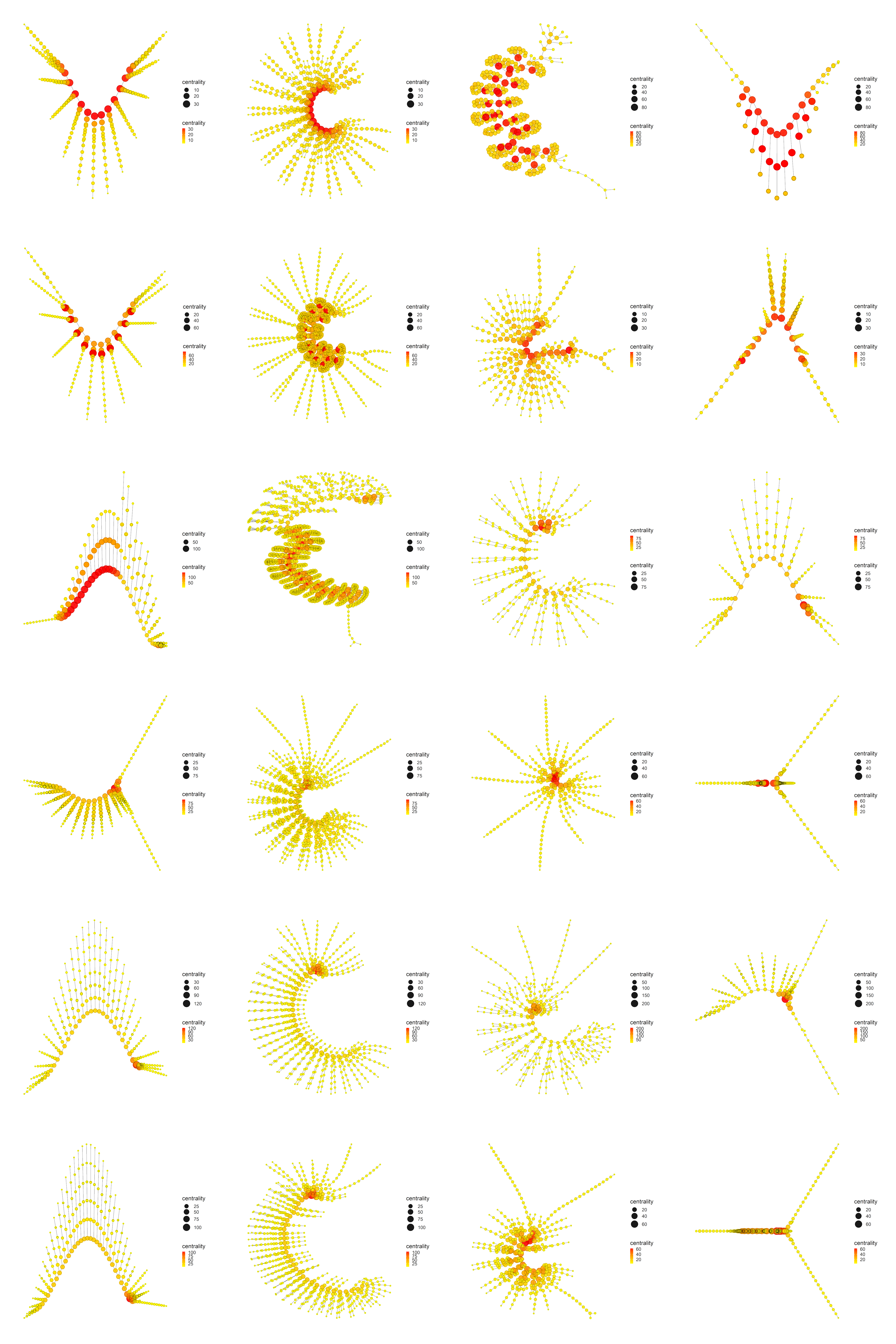

Supplement: Supplementary file 3 — Supplementary Figure S2. [file 41598_2023_51019_MOESM3_ESM.zip › Figure S2.tif]
