# Supplementary material for: A network analysis of early arthropod evolution and the potential of the primitive
Source: Sci Rep. 2024 Jan 4;14:503. doi: 10.1038/s41598-023-51019-x (PMC10766614; doi:10.1038/s41598-023-51019-x)
Supplement: Supplementary file 3 — Supplementary Figure S2. [file 41598_2023_51019_MOESM3_ESM.zip › Figure S2 (legend).docx]

# Figure S2

**Communicability centrality.** Display and spatialization of arthropod networks' Decay centrality based on two different layout algorithms (Kamada-Kawai (KK) in the two internal columns and MultiDimensional Scaling (MDS) in the two external columns). Arthropod networks are ordered according to the result of the hierarchical clustering. Right branch (column 3 (KK) and 4 (MDS), from row 1 to row 6): *Branchinecta*, *Waptia*, *Olenoides*, *Martinssonia*, *Nebalia*, *Lightiella*. Left branch (column 2 (KK) and 1 (MDS), from row 1 to row 6): *Yohoia*, *Canadaspis*, *Triops*, *Rehbachiella*, *Marrella*, *Speleonectes*. Color (from yellow to red) and size represent the centrality measure value of each node (see inset to the right of each network).
